# Nanocrystal Synthesis Derived Approach to Silver Bismuth Iodide Layered Double Perovskites with Aliphatic Amines: (C*_n_* H_(2*n*+1)_NH_3_)_4_AgBiI_8_

**DOI:** 10.1021/acs.chemmater.5c02845

**Published:** 2026-01-08

**Authors:** Pascal Rusch, Ann Mary Antony, Meenakshi Pegu, Meysoun Jabrane, Gabriele Saleh, Arghyadeep Garai, Aswin Asaithambi, Simone Lauciello, Sergio Marras, Serena De Negri, Pavlo Solokha, Liberato Manna

**Affiliations:** † Nanochemistry, 121451 Italian Institute of Technology, Via Morego 30, 16163 Genova, Italy; ‡ Departimento di Fisica, Politechnico di Milano, Edificio 8, Piazza Leonardo da Vinci, 20133 Milano, Italy; § Dipartimento di Chimica e Chimica Industriale, Università degli Studi di Genova, Via Dodecaneso 31, Genova 16146, Italy; ∥ Electron Microscopy, Italian Institute of Technology, Via Morego 30, 16163 Genova, Italy; ⊥ Materials Characterization Facility, Italian Institute of Technology, Via Morego 30, 16163 Genova, Italy

## Abstract

Lead-free iodide double perovskites are an interesting class of materials since they combine a relatively low toxicity (compared to the lead counterpart) with the small band gap typical of iodide-based perovskite structures. Their reported number is small due to their lower structural stability compared to the chloride and bromide analogues; hence, their synthesis is difficult. The structural constraints that limit stability, on the other hand, can be much relieved in layered organic–inorganic perovskites. Following this line of thought, we report here a successful fast precipitation route to iodide layered (C_*n*_H_(2*n*+1)_NH_3_)_4_AgBiI_8_ (*n* = 10, 12, and 14) double perovskites that borrow concepts from the synthesis of colloidal nanocrystals. X-ray diffraction studies revealed for these compounds a monoclinic crystal structure containing edge-sharing-alternating [AgI_6_] and [BiI_6_] octahedra. These materials have experimental band gaps of 2.1 eV, as also corroborated by theoretical calculations. We have also investigated their phase transitions by thermal analysis and temperature-dependent diffraction and found them to be similar to their lead-based layered perovskite counterparts.

## Graphical abstract

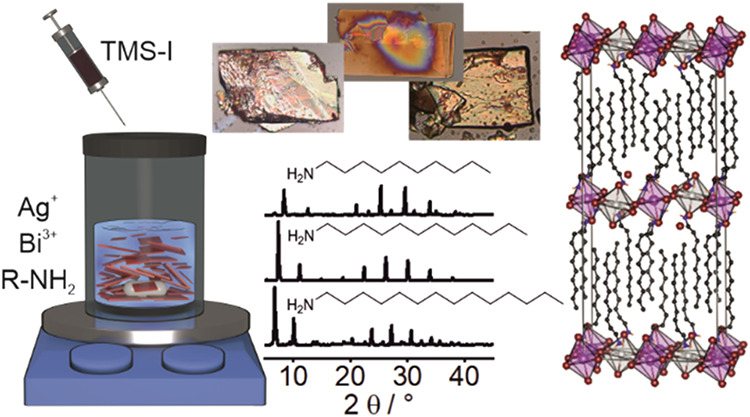

## Introduction

Lead halide perovskites have been widely investigated as highly promising materials, e.g., for photovoltaics,^1,2^ photodetectors,^3,4^ photocatalysts,^5–7^ and light emitters.^8–10^ In these applications, the iodide perovskites have gathered considerable interest due to their smaller band gap compared to their chloride and bromide analogues; hence, they are more suitable for photovoltaics.^11^ Unfortunately, the presence of toxic lead in these materials may represent a major concern for widespread applications.^2^ One of the possible approaches to circumvent this issue is the replacement of bivalent lead ions by two different cations, a monovalent cation like Ag^+^ and a trivalent cation like Bi^3+^, resulting in a double perovskite structure.^12,13^ Yet, while chloride and bromide double perovskites are fairly stable and have been widely investigated, the corresponding iodide counterparts are notably less stable, so that their investigation has advanced much less. For example, Cs_2_AgBiI_6_ is thermodynamically unstable and decomposes into CsAg_2_I_3_ and Cs_3_Bi_2_I_9_.^14^ On the other hand, the tight structural constraints imposed on the 3D perovskite structure are considerably relaxed in the case of 2D, layered perovskites (LPs).^15,16^ This has indeed led to the emergence of LPs with Ruddlesden–Popper^17^ and Dion–Jacobson^18^ architectures. In the context of our work, a silver bismuth layered double perovskite (LDP) combines the replacement of Pb^2+^ ions with the reduction in dimensionality, leading at once to less toxic and more structurally stable materials. Within these LDP materials, the ones incorporating iodide as anions are those that would benefit the most in terms of structural stability, derived from such a reduction in dimensionality. In addition, and similarly to their lead-based counterparts, iodide-based perovskites would have smaller band gaps, which, in principle, would make them better suited for photovoltaic applications.^19,20^

There are several examples in the literature of lead iodide LPs^21–23^ and silver bismuth bromide LDPs^24^ in which the organic cations are the protonated versions of simple aliphatic amines, i.e., CH_3_-(CH_2_)_n_-NH_2_. For lead iodide LPs, a wide range of linear chain amine-based compounds have been thoroughly synthesized and investigated regarding their temperature-dependent phase transitions,^25–27^ while, surprisingly, there are no reports on the use of these simple aliphatic amines and their respective ammonium ions as organic spacers in iodide LDPs. To date, the established strategies to prepare silver bismuth iodide LDPs have been limited to the use of templating organic ammonium ions as spacers that strongly interact with each other through π–π-stacking of aromatic groups or through hydrogen bonds with heteroatoms to induce the formation of a layered structure.^20,28^ The assumption in these approaches has been that the templating effect of these organic spacer ions aids the formation of such layered structures, as initially shown for vacancy-ordered metal iodide LPs.^29^ As a consequence of the alleged need for templating spacer molecules, the number of lead-free iodide LPs has remained relatively small to date, with all syntheses requiring the use of organic cations that include in their structure heteroatoms,^28,30–33^ aromatic functionalities,^34,35^ or both^20,28,30,36^ (these are summarized in Table S1).

In a recent work, a combination of high-throughput synthesis with machine learning algorithms has been used to screen several amines deemed favorable for the synthesis of iodide LDPs and has thus revealed numerous possible favorable combinations. Consistent with the results mentioned above, that work concluded that the use of “bulky”, structure-templating organic cations is necessary for the formation of iodide LDPs.^37^ Strikingly, none of these studies has included linear chain aliphatic amines, thus leaving the impression that these molecules are not amenable to LDPs.

In the present work, we overcome this apparent limitation, as we were able to synthesize iodide LDPs using simple aliphatic amines of various lengths. This was possible by devising an approach derived from the *hot-injection* synthesis,^38^ prevalent in the synthesis of colloidal nanocrystals. Similar to the case of nanocrystal synthesis, we rely on the fast injection of a highly reactive anion precursor, which triggers the rapid nucleation and growth of layered silver bismuth iodide crystals. When using the linear aliphatic amines decylamine, dodecylamine, and tetradecylamine, this process yields silver bismuth iodide LDPs (shown in Figure 1). These structures will be labeled C10, C12, and C14, respectively. Theoretical calculations indicate that these LDPs are semiconductors with an optical absorption edge around 2.1 eV and relatively flat bands, making a distinction between direct and indirect band gaps difficult. Also, similar to the Pb-based LPs, these LDPs exhibit distinct phase transitions with temperature.

**Figure 1:**
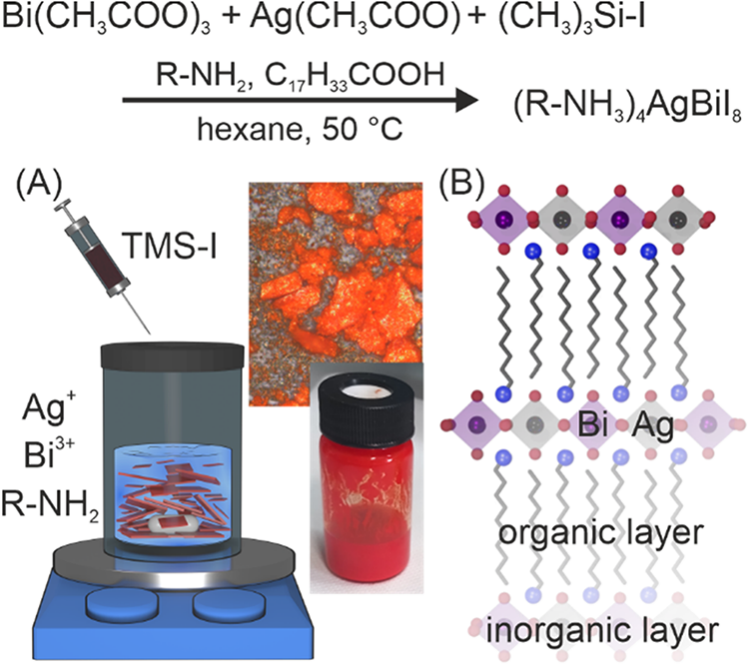
Rapid precipitation synthesis of the LDP samples. Schematic depiction (A) of the rapid precipitation method used in this work to synthesize layered silver bismuth double perovskites and photographs of the synthesized microcrystals dispersed in solvent (vial) after reaction and as dried powder, and sketch (B) of their structure.

## Experimental Section

### Chemicals

Bismuth acetate (99.99%), silver acetate (99.99%), iodotrimethylsilane (97%, stored over Cu filings), oleic acid (90%), 1-octadecene (90%), hexane (99%), decylamine (99%), dodecylamine (97%), tetradecylamine (95%), 4-fluorophenethylamine (97%), chlorotrimethylsilane (98%), bromotrimethylsilane (97%), diethyl ether (99.8%), Hellmanex, acetone (99.5%), 2-propanol (99.8%), and *N,N*-dimethylformamide (DMF, 99.8%) were purchased from Sigma-Aldrich and used without further purification. Silane halide precursors are stored in an N_2_-filled glovebox (iodotrimethylsilane in the glovebox freezer at −8 °C) and handled under standard air-free techniques due to the rapid reaction of silanes with moisture and oxygen. After synthesis, all samples were handled and stored under ambient atmosphere.

### Preparation of the Bismuth Oleate Solution

Bismuth acetate (1 mmol, 386 mg) is dissolved in a mixture of 1 mL of oleic acid and 1 mL of 1-octadecene by heating to 120 °C under vacuum for 30 min, followed by 30 min at 150 °C under a nitrogen atmosphere. The transparent, colorless solution is left to cool to room temperature and stored under nitrogen.

### Preparation of the Silver Bismuth Iodide LDPs

In a vial, 0.1 mmol of silver acetate, 0.5 mmol of the targeted amine, and 5 mL of hexane are mixed, and 0.2 mL of the above-mentioned bismuth oleate solution is added (corresponding to 0.1 mmol of Bi). The reaction mixture is stirred intensely while injecting 0.1 mL of iodotrimethylsilane with a syringe (or the corresponding other halide trimethylsilane). An immediate precipitation of a deep red solid can be observed. This solid is washed three times by centrifugation at 4000 rpm and redispersion in hexane using a vortexer. The solid is stored under hexane under ambient conditions. For recrystallization, highly concentrated solutions were required; this synthesis procedure could be scaled up by a factor of 4 to produce ca. 800 mg of the product in a single synthesis.

For recrystallization, acetone is added to the hexane dispersion dropwise until dissolution of the precipitate to form a clear, deep orange solution. The acetone/hexane solvent mix is allowed to evaporate slowly through a perforated parafilm in a saturated hexane atmosphere.

### Preparation of Thin Films

Glass slide substrates for spin-coating were cleaned before deposition by consecutive immersion and ultrasonication for 20 min each in Hellmanex solution, Milli-Q water, acetone, and isopropanol. The substrates were dried under a nitrogen flow. To prepare thin films of the double perovskite samples, the dispersions were centrifuged again and the supernatant discarded. The powder sample was washed two additional times with diethyl ether and dried completely. 400 mg of the resulting powder was dissolved in 0.2 mL of DMF to result in a ca. 0.2 M concentration. These solutions were used for spin-coating the material on the substrates mentioned above by a two-step process of 10 s at 1000 rpm and 50 s at 10,000 rpm, depositing 40 μL of solution.

### Optical Characterization

Absorption spectroscopy of spin-coated sample films on glass slides was performed by using an Agilent Cary5000 spectrometer equipped with a DRA-2500 integrating sphere. The samples were mounted in a slide holder in the center position with an incident angle of 10°. Low-temperature photoluminescence spectroscopy measurements were performed using a Renishaw inVia Reflex Raman microscope with 457 nm laser excitation focused on the sample via a 50× long working distance objective lens. The PL light is collected via the same objective, and a 600g/mm coarse grating was used for the PL measurements. An excitation power of ∼1 mW was used. The sample was kept in a Linkam flow cryostat chamber.

### Electron Microscopy

Scanning electron microscopy images of the sample powders and crystals were collected using a Zeiss GeminiSEM 560 instrument, which was also used for elemental analysis by energy-dispersive X-ray spectroscopy. The powder samples were drop-cast onto Si wafers as diluted dispersions in hexane and dried under ambient conditions.

### Optical Microscopy

Light microscope images of the powders and crystals were acquired by using a ZETA optical profilometer Zeta-20.

### X-ray Diffraction (XRD)

Powder X-ray diffraction (p-XRD) patterns were collected using a third-generation Empyrean diffractometer (Malvern-PANalytical, Westborough, MA) equipped with a 1.8 kW Cu Kα X-ray tube operating at 45 kV and 40 mA, automated prefix iCore-dCore optical modules for the incident and diffracted beam paths, and PIXcel3D area detector. Temperature-dependent measurements were carried out using an Anton Paar TTK600 chamber, under N_2_ flow (5Nl/min). Powdered samples were finely ground in an agate mortar and flattened on the holder before the measurement, while the dispersions of microcrystals were drop-cast from a hexane solution. In both cases, a zero-diffraction silicon sample holder was used. HighScore Plus 5.3 software from Malvern-PANalytical was used for data analysis.

### Single-Crystal X-ray Diffraction

Translucent red, plate-shaped crystals of the (C*_n_* H_(2*n*+1)_NH_3_)_4_AgBiI_8_ (*n* = 10, 12, 14) compounds were selected under a light optical microscope, glued to glass fibers, and mounted on the goniometer. A three-circle Bruker D8 QUEST diffractometer using Mo Kα radiation (λ = 0.71076 Å) and equipped with a PHOTON III photon counting detector was used for measurements. The data collection strategies, elaborated using APEX5 software,^39^ covered the reciprocal space up to a maximum θ of about 28° (resolution of ca. 0.75 Å). All data were integrated with SAINT V8.40B,^40^ and a multi-scan absorption correction using SADABS 2016/2 was applied.^41^ The structure was solved by the intrinsic phasing method with SHELXT 2018/2 and refined by full-matrix least-squares methods against F^2^ using SHELXL-2019/2.^42,43^ All non-hydrogen atoms were refined with anisotropic displacement parameters. All C-bound hydrogen atoms were refined isotropically on calculated positions using a riding model with their U_iso_ values constrained to 1.5 times the U_eq_ of their pivot atoms for terminal sp^3^ carbon atoms and 1.2 times for all other carbon atoms. Crystallographic data for the structures reported in this paper have been deposited with the Cambridge Crystallographic Data Centre,^44^ and they can be obtained free of charge from the Cambridge Crystallographic Data Centre via www.ccdc.cam.ac.uk/structures under the following codes: (C_10_H_21_–NH_3_)_4_AgBiI_8_-2491406, (C_12_H_25_–NH_3_)_4_AgBiI_8_-2366438, and (C_14_H_29_–NH_3_)_4_AgBiI_8_-2491405. Selected crystallographic information is reported in the Supporting Material. The structures were visualized using the VESTA software package.^45^

### Thermal Analysis

Differential scanning calorimetry (DSC) was performed on a Waters-TA Instruments Discovery DSC 250 with Discovery Refrigeration Cooling System RSC90 in aluminum nonhermetic TZero pans under nitrogen atmosphere from −90 to 130 °C at a rate of 5 °C/min. Before the DSC measurement, thermogravimetry was performed to confirm that no mass loss was happening in the investigated temperature range.

### Computational Details

Density functional theory (DFT) calculations were carried out using the Vienna Ab initio Simulation Package (VASP).^46–48^ The interaction between valence electrons and ionic cores was treated with the projector augmented-wave (PAW) method, and a plane-wave cutoff energy of 500 eV was used for the C10, C12, and C14 structures.^49,50^ For the exchange–correlation functional, we adopted the Perdew–Burke–Ernzerhof (PBE) form of the generalized gradient approximation (GGA).^51^ The atomic coordinates of all three structures were taken directly from experimental crystallographic data, and no relaxation was performed. The effect of spin–orbit coupling (SOC) was investigated separately, (re)­calculating the density of states (DOS) and band gap for C12.

## Results and Discussion

Silver bismuth iodide LDPs were synthesized by a rapid precipitation. This method consists of solubilizing the metallic ions using oleic acid and the desired amine in organic solvents, followed by the addition of trimethylsilyl iodide (schematically shown in Figure 1A), as detailed in the Experimental Section. This resulted in the immediate precipitation of a bright red or orange product (Figure 1A). The initial rapid precipitation, with the injection of the iodide precursor, aids in avoiding the formation of side products and facilitates a fast and simple synthesis of such structures.

The layered structure of the product obtained using long-chain amines (decylamine, dodecylamine, and tetradecylamine) is clearly evident from the powder X-ray diffraction (p-XRD) patterns. Dominant basal reflections (*h*00) appear with uniform spacing, corresponding to the interlayer distance, and are indicative of a preferred orientation of the crystallites along the stacking direction (see Figure 2). By scanning electron microscopy (SEM), the powders appear as polydisperse microcrystals a few μm in size (Figure S1). The elemental analysis of these powders by energy-dispersive X-ray spectroscopy (EDX) reveals an Ag:Bi:I ratio of ca. 1:1:8 for these samples (see Table S2 and Figure S5–7 for details), which matches the expected composition of an LDP. On the other hand, using shorter carbon chain amines, i.e., butylamine and hexylamine, the initial characterization did not indicate a layered structure (see Figure S2), and a spatially resolved elemental analysis showed a mixture of bismuth and silver iodides (see Figures S3, S4 and Table S2). Therefore, the work was focused on the more promising powder samples with decylamine (C10), dodecylamine (C12), and tetradecylamine (C14), on which a structural solution and further characterization were pursued. Accordingly, slow crystallization from a solvent-antisolvent mixture gave larger single crystals of a few hundred micrometers (Figures 2A and S8–S10). Therefore, it was possible to completely determine the crystal structure of the three compounds by single-crystal X-ray diffraction, confirming their 2D nature: single inorganic layers of alternating corner sharing Ag–I and Bi–I octahedra are separated by organic layers of the corresponding amines (as shown in Figure 3).

**Figure 2:**
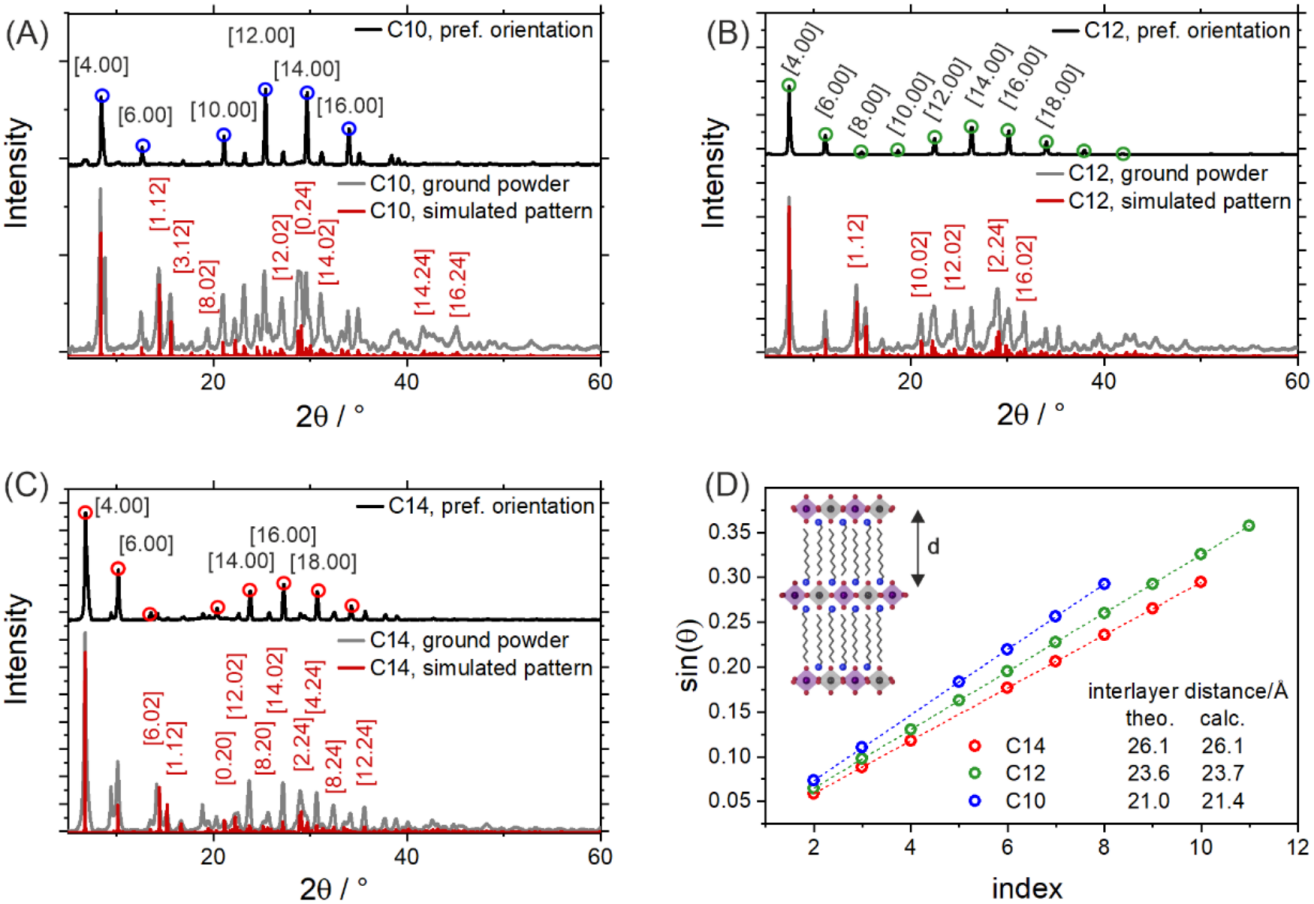
Powder diffractograms of drop-cast (A) C10, (B) C12, and (C) C14 products (black trace with the prominent [*h.*00] basal reflections marked) and comparison to the diffractogram of the dried and ground powder (gray trace) and to the calculated diffraction pattern (red trace with selected additionally visible [*h.kl*] reflections marked) based on the structural model. (D) Determination of the interlayer distance (sketched in the inset) from the basal reflections.

**Figure 3:**
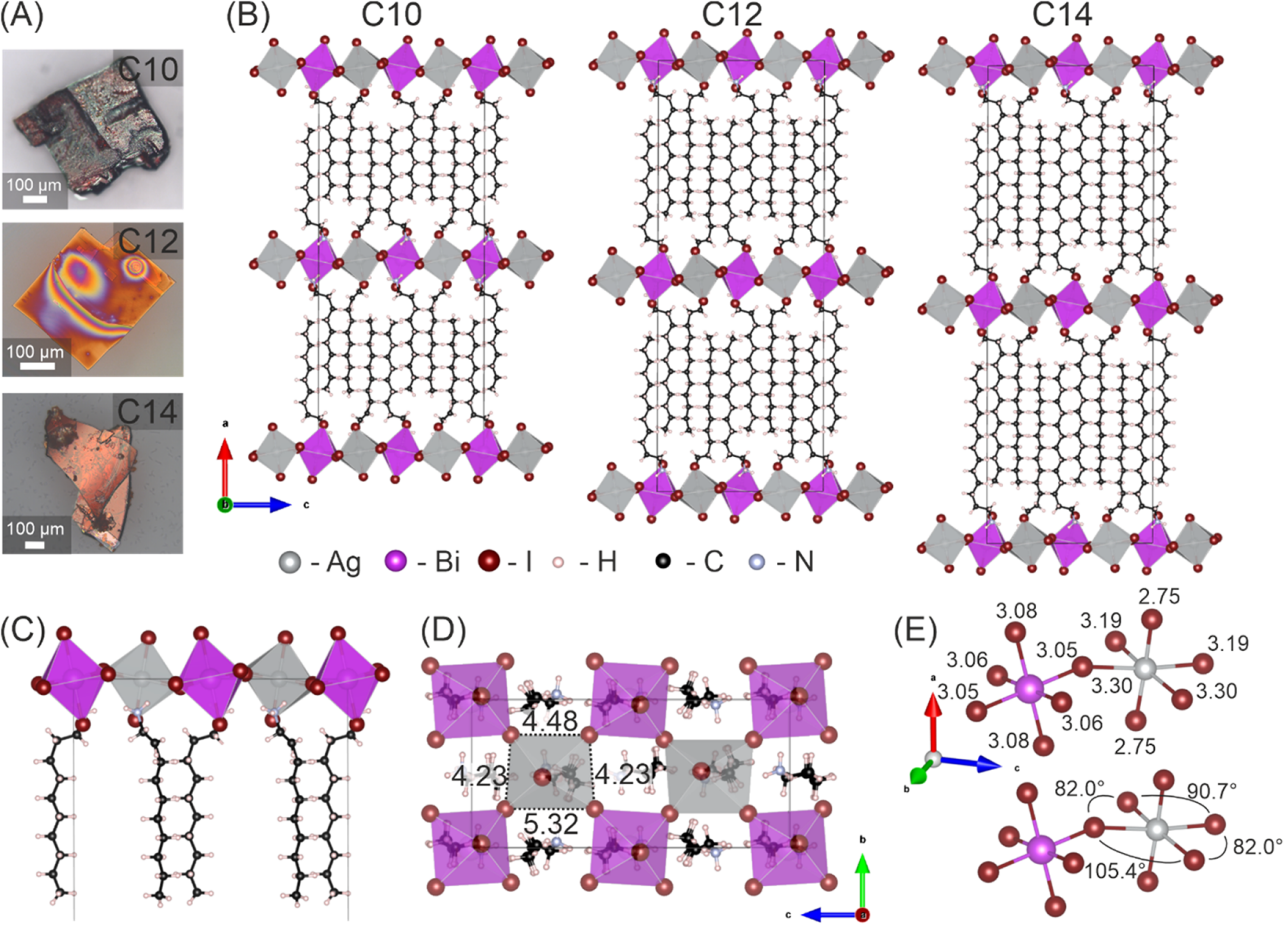
Structure of silver bismuth iodide LDPs with linear chain amines. (A) Single crystals of samples grown using decylamine (C10), dodecylamine (C12), and tetradecylamine (C14) in optical microscopy (top to bottom) and (B, left to right) their room-temperature structures viewed along the *b*-axis. (C) Enlarged image of the C10 structure highlighting the amine groups’ positioning to maximize their interaction with the iodide ions. (D) Crystal structure of the C10 perovskite viewed along the *a*-axis. (E) Selected distances (Å) between metal ions and coordinating iodide ions in the inorganic octahedra and angles within these octahedra for C10.

All compounds crystallize in the same monoclinic space group *C*2/*c* (more details are listed in Table 1) and show a very similar architecture. This explains why the cell metrics are also similar, except for the *a* parameter, which correlates with the length of the olefin chain of the organic spacer. Accordingly, it changes from 42.2604 Å (C10) to 47.3629 Å (C12) and 52.494 Å (C14). A rough estimate for the *a* parameter can be derived from the length of the aliphatic amine^52–54^ and the average length of bismuth–iodine bonds^55^

d=2×(Bi−Ibondlength)+(lengthofaliphaticaminewithncarbons)

d=2×3.1Å+(2Å+1.28Å×n)

**Table 1:** Crystallographic Details of the Resolved Structures of the C10, C12, and C14 AgBiI Samples at Room Temperature

CCDC number	2491406	2366438	2491405
Formula	(C_10_H_21_–NH_3_)_4_AgBiI_8_	(C_12_H_25_–NH_3_)_4_AgBiI_8_	(C_14_H_29_–NH_3_)_4_AgBiI_8_
Formula weight [g mol^–1^]	1965.25	2077.46	2189.67
Crystal size [mm^3^]	0.03 × 0.08 × 0.1	0.01 × 0.12 × 0.25	0.04 × 0.19 × 0.21
Crystal color	translucent intense red		
Crystal shape	plate like		
Crystal system	monoclinic		
Space group (number), Z	*C*2/*c* (15), 4		
*a* [Å]	42.2604(13)	47.359(4)	52.494(3)
*b* [Å]	8.4641(3)	8.4030(8)	8.4011(5)
*c* [Å]	18.2342(6)	18.2429(16)	18.2415(10)
β [°]	90.1760(10)	90.197(3)	90.282(2)
Volume [Å^3^]	6522.3(4)	7259.9(11)	8044.6(8)
ρ_calc_ [gcm^–3^]	2.001	1.901	1.808
μ [mm^–1^]	6.807	6.121	5.529
*F*(000)	3672	3928	4184
2θ range [°]	4.86 to 61.05 (0.70 Å)	4.78 to 56.70 (0.75 Å)	4.72 to 56.85 (0.75 Å)
Index ranges	–60 ≤ *h* ≤ 60	–62 ≤ *h* ≤ 62	–70 ≤ *h* ≤ 69
	–12 ≤ *k* ≤ 12	–11 ≤ *k* ≤ 11	–11 ≤ *k* ≤ 11
	–26 ≤ *l* ≤ 26	–23 ≤ *l* ≤ 24	–24 ≤ *l* ≤ 24
Reflections collected	168657	3488	47270
Independent reflections	9950	9044	10090
	*R*_int_ = 0.1143	*R*_int_ = 0.0833	*R*_int_ = 0.1276
	*R*_sigma_ = 0.0619	*R*_sigma_ = 0.0408	*R*_sigma_ = 0.0689
Completeness to θ = 25.243°	99.9	100.0	99.9
Data/Restraints/Parameters	9950/0/250	9044/0/286	10090/0/322
Goodness-of-fit on *F* ^2^	0.978	1.165	1.093
Final *R* indexes [*I* ≥ 2σ(*I*)]	*R*_1_ = 0.0426	*R*_1_ = 0.0695	*R*_1_ = 0.0591
	w*R*_2_ = 0.0778	w*R*_2_ = 0.1563	w*R*_2_ = 0.1512
Final *R* indexes [all data]	*R*_1_ = 0.1220	*R*_1_ = 0.0990	*R*_1_ = 0.1140
	w*R*_2_ = 0.1015	w*R*_2_ = 0.1670	w*R*_2_ = 0.1793
Largest peak/hole [eÅ^–3^]	1.43/–0.79	1.88/–1.37	2.34/–1.97

In this case, the cell contains two of these slabs, and the parameter *a* corresponds well to the theoretical assumption with C10:2*d* = 2 × 21.0 = 42.0 ≈ 42.2604, C12:2*d* = 2 × 23.6 = 47.2 ≈ 47.3629, and C14:2*d* = 2 × 26.1 = 52.2 ≈ 52.494. These values also correspond well with those extracted from p-XRD (Figure 2D) on drop-cast samples. The p-XRD of the dried and ground initial powders was also measured to minimize the influence of the (*h*00) basal reflections due to the preferential orientation. The resulting diffraction patterns match well with the calculated ones (Figure 2A–C). Importantly, this leads us to conclude that the LDP structure does not simply form during the recrystallization but is present from the initial rapid precipitation synthesis.

In the inorganic layer, the connected Ag and Bi octahedra are tilted with respect to each other (Ag–I–Bi angle of 167.3°). The [BiI_6_] octahedra within these layers are almost symmetrical, with all I–Bi–I angles close to 90° and only a minor difference between the Bi–I bond lengths in the axial and equatorial positions. On the other hand, the [AgI_6_] octahedra are strongly distorted. The Ag–I bond lengths are considerably longer in the equatorial direction than in the axial direction (by ca. 0.5 Å; see Figures 3E and S11). The two different equatorial bond lengths are adjacent: this results in the octahedral plane being a trapezoid with the sides defined by the I–I distances (as visualized in Figure 3D as a top-down view, see also Figure S12). The exact bond lengths and angles differ only minutely between the C10 structure and its respective C12 and C14 analogues. Within one row of [AgI_6_] octahedra, all of the mentioned trapezoids are oriented in the same direction toward the *b*-axis. The next respective rows of [AgI_6_] octahedra in the *c* direction show a reversed orientation of these trapezoids (see also Figure 3D). Looking at the organic spacer, two observations can be made. (1) They appear to occupy two different positions, one in which all carbons are fairly well localized and a second one in which the first three carbons adjacent to the amine group display a much higher flexibility in position (see Figure S13). This difference is likely caused by the octahedral tilt to maximize the interaction between the ammonium cation and the iodide anions. Additionally, the strong asymmetry in the Ag octahedra results in a larger space available for the amine next to the “long” sides of the trapezoid described above and a smaller space next to the “short” sides (visualized in Figure 3D,E). (2) Considering the long, flexible carbon chain of the amines, the atomic positions in this chain are remarkably defined (see the probability ellipses in Figure S13). This suggests high order within the interjecting alkyl chains. Organic moieties that interact strongly, e.g., containing pyrene groups forming π–π-stacks^35^ or polar heteroatoms like 4-iodobutanamine,^33^ inducing H-bond formation, have been shown to promote the formation of layered iodide double perovskite structures. Here, we assume a similar effect caused by interdigitated alkyl chains of the aliphatic amines. While the strong ionic binding in the inorganic layer is the likely driving force behind the formation and precipitation of the product structures, the van der Waals interactions between the alkyl chains can serve to direct an evolution of the layered structure.

Layered lead iodides, having structures comparable to those described for the double perovskites herein, have been reported to exhibit multiple distinct phase transitions with temperature.^25–27^ To investigate potentially similar behavior, we performed differential scanning calorimetry (DSC) and temperature-dependent p-XRD measurements (discussed in detail in the Supporting Information and shown in Figures 4 and S14). Thermal events at similar temperatures compared to the literature analogues, and a prominent change of ca. 2 Å in interlayer distance above 50 °C (C10) and above 75 °C (C12) are evident from p-XRD. These results indicate a likely similar behavior between the silver bismuth iodide LDP structures reported here and their analogue lead iodide LP structures reported by Billing and Lemmerer.^25,26^ This further supports the hypothesis of the aliphatic amine exerting an influence on the structure of the LDP.

**Figure 4:**
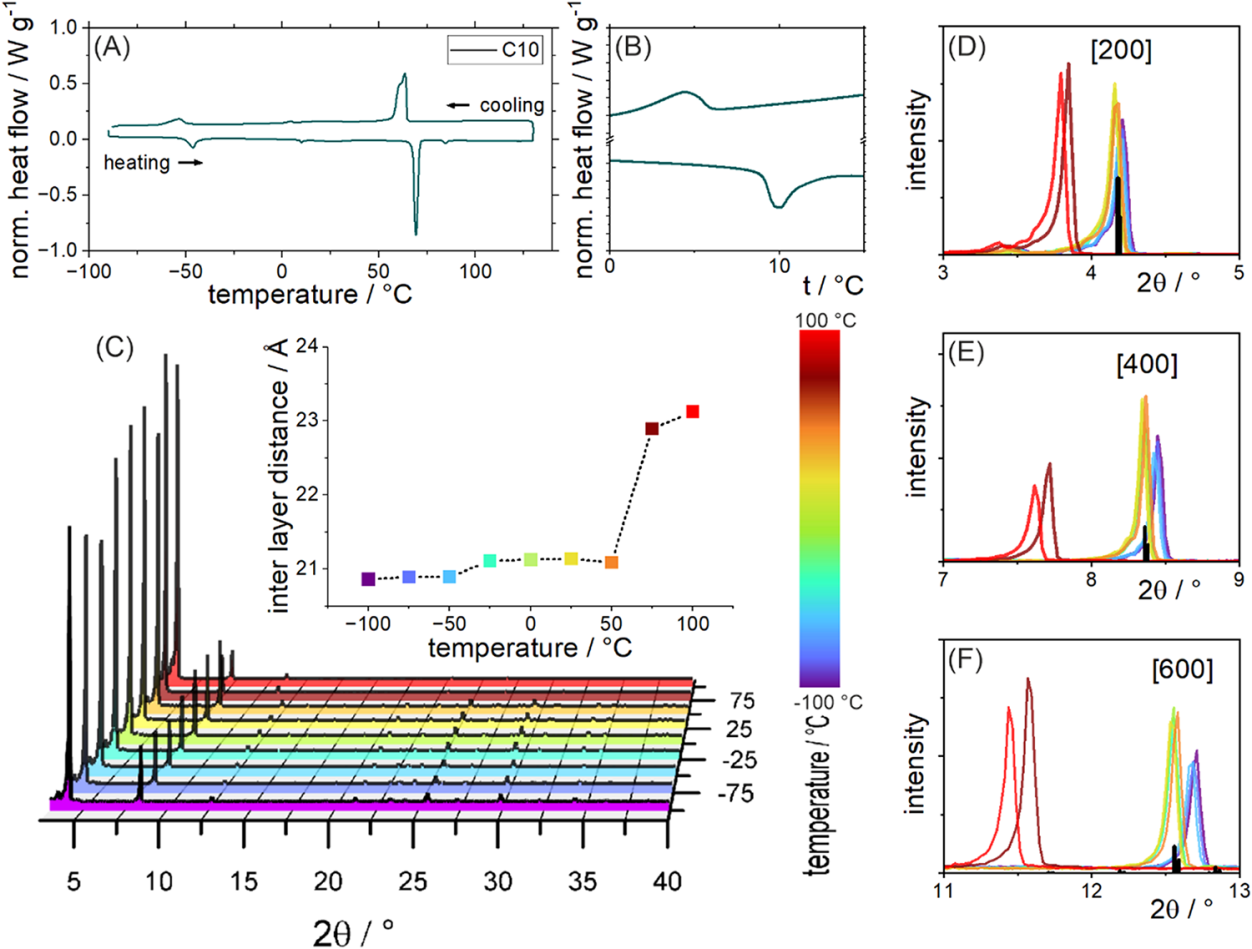
DSC of the powdered (A) C10 silver bismuth iodide LDPs with a region around 10 °C shown separately (B) to visualize the small thermal event there. (C) p-XRD measured at different temperatures of the C10 sample, with the interlayer distances extracted from the [*h*00] basal reflections at different temperatures for the C10 and C12 samples. The changes at the first three observed major reflections corresponding to (D) [200], (E) [400], and (F) [600] are shown in detail, along with the reference positions calculated from the resolved single-crystal structure.

To evaluate the optical properties, the powder samples were dissolved in polar solvents and spin-coated onto borosilicate glass slides (light and electron microscopy images of an exemplary film are shown in Figure S15). The identity of these thin films was confirmed by XRD (Figure 5A). It is apparent that they correspond to the respective structures observed in powder and single crystals, with an even more prominent influence of preferential orientation in the thin film, which is expected. The optical properties of the three samples, C10, C12, and C14, were remarkably similar. All three samples show an absorption edge around 580 nm (Figure 5B), matching their red color. The optical spectra of the three materials were displayed in a Tauc plot (see Figure S16) to extract their band gap, yielding values of 2.01 eV (C10), 2.05 eV (C12), and 2.01 eV (C14). An additional absorption feature at a higher energy is visible around 450 nm. These results are similar to the ones already reported for comparable materials,^20,28,30–32,34,37^ with only a minor influence of chain length of the organic cation, which again is expected since the optical absorption of the materials is dominated by the inorganic layer of Ag and Bi octahedra. LDPs similar to the ones prepared in this work have been claimed to have a direct band gap;^20,28^ therefore, the emission properties of the LDPs synthesized by us were investigated as well. At room temperature, no emission signal could be observed. When cooling the samples to liquid nitrogen temperature (below the low-temperature phase transition temperature discussed above), a broad photoluminescence with maxima around 575 nm, 625nm, and 660 nm could be observed for the C10 sample (Figure S17A). In the case of the C12 sample, any emission was hardly measurable even below −100 °C (Figure S17B). The low intensity and broadness of the emission hint at either strong trapping inhibiting recombination or an indirect band gap.

**Figure 5:**
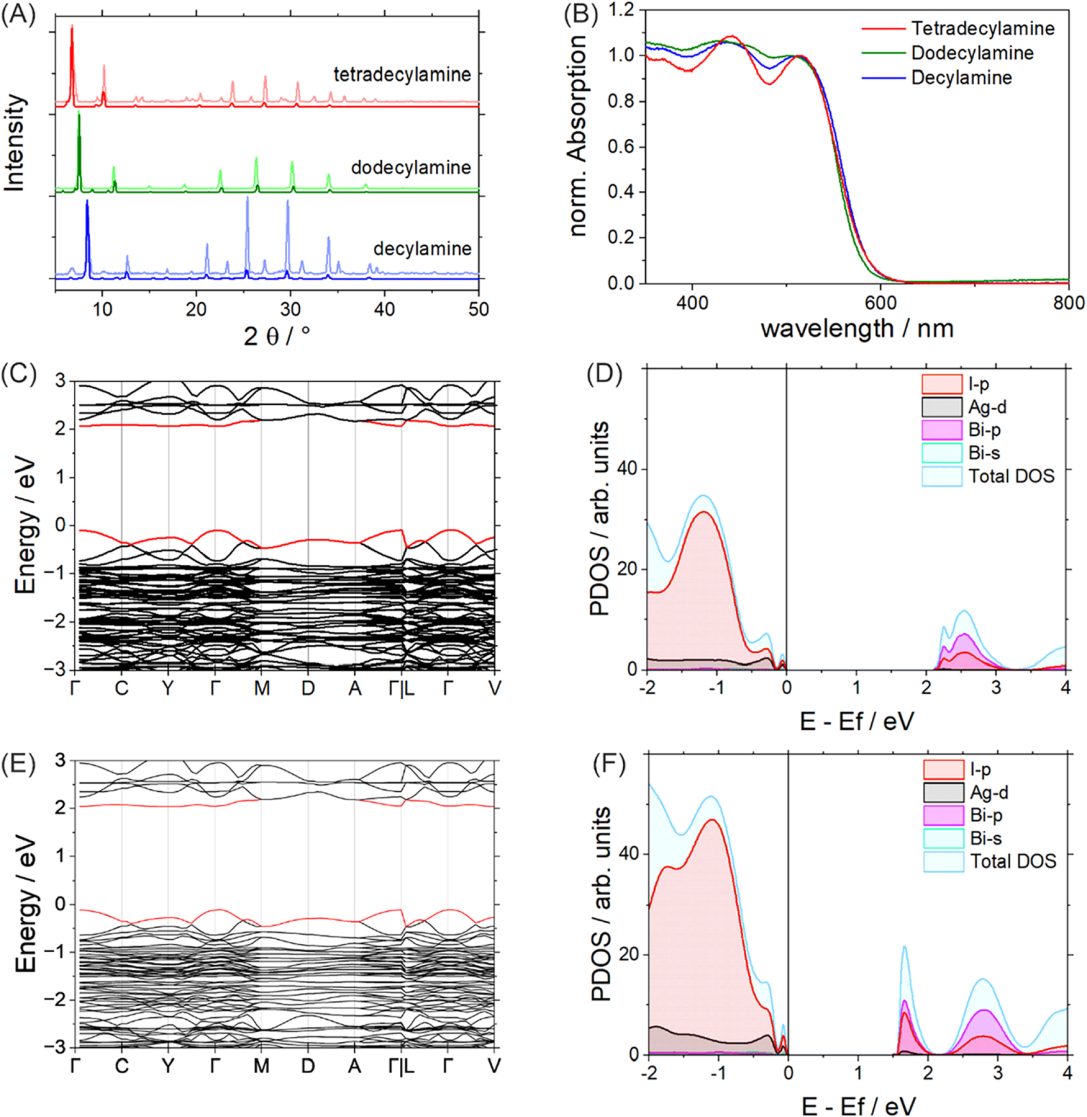
Electronic properties of the layered AgBi iodide double perovskites are determined by optical characterization and theoretical calculations. XRD measurements (A) comparing thin film (bold trace) and drop-cast powder dispersions (light trace), and room-temperature absorption spectra (B) of C10, C12, and C14 AgBiI_8_. Calculated band structures of (C) (C_10_H_21_NH_3_)_4_AgBiI_8_ and (E) (C_14_H_29_NH_3_)_4_AgBiI_8_. Density of states was calculated without (D) spin–orbit coupling and with (F) spin–orbit coupling of­(C_10_H_21_NH_3_)_4_AgBiI_8_.

To further elucidate the electronic properties of the materials, their band structure was modeled by density functional theory simulations adopting the PBE functional (as described in the Experimental Section). The band structures of C10, C12, and C14, shown in Figures 5C,E and S18, are quite similar, with calculated band gaps of 2.15 eV, 2.12 eV, and 2.15 eV, respectively, in fairly good agreement with our experimental data. As observed in previous studies,^19,27,29–31,34,36^ the conduction band minimum (CBM) mainly originates from unoccupied Bi-*p* and I-*p* orbitals, while the valence band maximum (VBM) is composed of Ag-*d* and I-*p* states, as seen in the projected density of states (PDOS) shown in Figure 5D. In all three materials, the conduction band shows multiple nearly degenerate minima at different high-symmetry points in the Brillouin zone, resulting in a relatively flat CBM region (red line). This indicates a high effective mass of the excited electrons, thus suggesting poor electronic conductivity. Moreover, the flatness of the bands makes it difficult to clearly classify the band gap as direct or indirect. For example, in C14 (Figure 5E), the CBM and VBM are located at the V point and the Γ point, respectively, but the energy difference from the closest direct transition is only 0.01 eV. This direct–indirect gap difference is lower than the room-temperature thermal energy, thus making the direct gap easily accessible.

The inclusion of spin–orbit coupling (SOC) was shown in previous studies to produce a clear splitting of the Bi 6p orbitals, particularly lowering the 6p_1_/_2_ states.^20,28^ Accordingly, in our system, SOC splits the conduction band states, lifting the near-degeneracy of the Bi-p states. Notably, the character of CBM and VBM (see above) is unaffected by SOC. In particular, the conduction band remains flat near its minimum (Figure 5F).^20,28,31^ However, we note that the introduction of SOC worsens the agreement with the experimental band gap. Indeed, given the large size of the studied systems, to simulate their band gap, we exploited the well-known error compensation between the adoption of the generalized gradient approximation (PBE functional) and the neglection of SOC. Nonetheless, our investigation on the effect of SOC confirmed the electronic structure features observed adopting the PBE functional without SOC.

To understand the influence of our synthesis approach, different parameters were varied. A controlled slow injection of iodotrimethylsilane over 5 and 60 min was performed to understand the formation of the product. This caused a more gradual increase in iodide concentration compared with the rapid injection. In all three reactions, independent of the injection speed, the main product is the described LDP structure (Figure S20). A second row of experiments was performed to understand the influence of the temperature. Here, the reaction temperature at which the iodide precursor was injected was varied between 0 and 50 °C. A change in injection temperature had no notable influence on the product phase (see Figure S21). Lastly, a comparative reaction was conducted using the established approach of slow crystallization from hydroiodic acid. Specifically, the combination of silver and bismuth iodide with decylamine in hydroiodic acid produced the same LDP structure described herein as the main product (Figure S22). Overall, the introduced silver bismuth iodide LDPs forming largely independently of these synthesis parameters indicate them to be the thermodynamically stable product. However, when using a synthesis with slow product formation, additional reflections appear at 5–10° 2θ in the p-XRD (Figures S20 and S22). This indicates the presence of contaminant side products, thus complicating product recovery and purification in these cases.

Considering the shown phase transitions close to room temperature and the general susceptibility of halide perovskite structures to moisture and ambient atmosphere, the structures showed appreciable stability, with the p-XRD pattern clearly matching the initial product phase. Nonetheless, a partial loss in crystallinity was apparent after ambient storage for one year (see Figure S23).

The generality of our synthesis approach was assessed by synthesizing the already reported 4-fluorophenethylamine (4-FPEA) silver bismuth iodide.^20^ A clear match of the product using FPEA to the structure reported in the literature is apparent from the powder XRD (Figure S24). This indicates that our nanocrystal-derived synthesis approach is viable for other LDP structures as well. It was equally possible to substitute the used iodide precursor, iodotrimethylsilane, with analogous counterparts for other halides to form the respective layered silver bismuth bromides and chlorides (Figure S26). As expected for halide perovskites, the introduction of other halides drastically changes the optical properties of the resulting materials, the absorption edge shifting from 580 nm (for the iodide) to 490 nm (for the bromide) to 420 nm (for the chloride) in the case of the dodecylamine-containing structures investigated here (Figure S27). This change is also apparent in the color of the product microcrystals, with the iodide being red, the bromide yellow, and the chloride colorless.

## Conclusion

In this work, we introduced an alternative synthesis route to silver bismuth iodide layered double perovskites, adapted from nanocrystal hot-injection procedures. Using this route, a set of layered silver bismuth iodides with aliphatic amines of different carbon chain lengths as organic spacers could be prepared. The powdered samples could be employed as precursors for further crystal growth to yield larger single crystals and thin films for characterization. This enabled the product structures to be determined and refined by single-crystal diffraction. The temperature-dependent characterizations revealed strong similarities between the layered double perovskites discussed here and previously reported layered perovskites containing the same amine. This reinforces the hypothesis of aliphatic amines acting as strong structure-directing agents in this case. While for structure and phase transitions an influence by the layers of interdigitated organic amines is observable, from calculations and optical spectroscopy, their optical and electronic properties appear to be solely dependent on the inorganic layer. This fast injection synthesis was finally expanded to yield other literature-known similar compounds as well as the additional halides (Br and Cl), demonstrating a broader applicability of this approach.

## Supplementary Material



## Notes

Elemental characterization, additional structural details, temperature-dependent measurements for C12, low-temperature PL measurements of C10, C12, and C14, and characterization of (4-FPEA) silver bismuth iodide, C12 silver bismuth bromide, and C12 silver bismuth chloride (PDF)

## #

Pascal Rusch, Department of Chemistry, University of Basel, Mattenstrasse 22, 4058 Basel, Switzerland
