# Supplementary material for: Nanocrystal Synthesis Derived Approach to Silver Bismuth Iodide Layered Double Perovskites with Aliphatic Amines: (C n H(2n+1)NH3)4AgBiI8
Source: Chem Mater. 2026 Jan 8;38(2):900–9. doi: 10.1021/acs.chemmater.5c02845 (PMC12854703; doi:10.1021/acs.chemmater.5c02845)
Supplement: Supplementary file 1 [file cm5c02845_si_001.pdf]

Supporting Information: Nanocrystal Synthesis Derived Approach to Silver Bismuth Iodide Layered Double Perovskites with Aliphatic Amines: $(C_nH_{(2n+1)}NH_3)_4AgBiI_8$

*Pascal Rusch,[a†] Ann Mary Antony,[a,b] Meenakshi Pegu,[a] Meysoun Jabrane,[a] Gabriele Saleh,[a] Arghyadeep Garai,[a,c] Aswin Asaithambi,[a] Simone Lauciello,[d] Sergio Marras,[e] Serena De Negri,[c] Pavlo Solokha,[c] Liberato Manna[a]**

[a]Nanochemistry, Italian Institute of Technology, Via Morego 30, 16163 Genova, Italy

[b]Departimento di Fisica, Politechnico di Milano, Edificio 8, Piazza Leonardo da Vinci, 20133 Milano, Italy

[c]Dipartimento di Chimica e Chimica Industriale, Università degli Studi di Genova, Via Dodecaneso 31, Genova, 16146 Italy

[d]Electron Microscopy, Italian Institute of Technology, Via Morego 30, 16163 Genova, Italy

[e]Materials Characterization Facility, Italian Institute of Technology, Via Morego 30, 16163 Genova, Italy

Table S1. Known examples of $(Amine)_4AgBiI_8$ layered double perovskites.

**Amine**		**Synthesis method**	**Band gap**	**Reference**
**4-fluorophenethylamine**	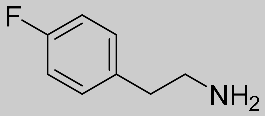	Slow crystallization from HI	2.16 eV	[19]
**5,5′-diylbis-(aminoethyl)-[2,2′-bithiophene]**	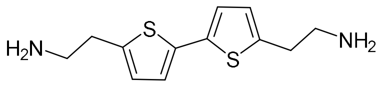	Slow crystallization from HI	2.0 eV	[27]
**4,4-difluoropiperidine**	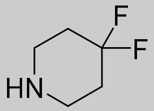	Slow crystallization from HI	1.93 eV	[35]
**4-aminomethylpiperidine**	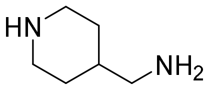	Slow crystallization from HI	1.27 eV	[29]
**4-aminomethylpyridine**	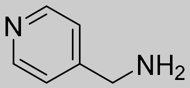	Slow crystallization from HI	1.44 eV	[29]
**4-iodobutylamine**	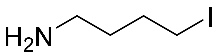	Slow crystallization from HI	2.0 eV	[32]
**3-iodopropylamine**	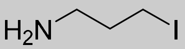	Slow crystallization from HI	1.87 eV	[30]
**3-(aminomethyl) pyridine**	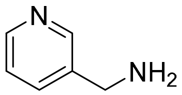	Slow crystallization from HI	1.86 eV	[33]
**naphthalene-*O*-ethylamine**	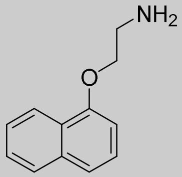	Crystallization from polar organic solvents (DMF/DMSO)	2.19 eV	[34]

**naphthalene-*O*-propylamine**	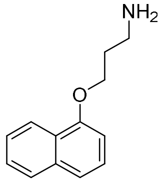	Crystallization from polar organic solvents (DMF/DMSO)	2.18 eV	[34]
**pyrene-*O*-ethylamine**	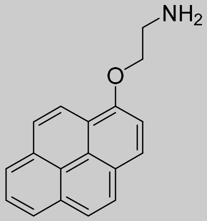	Crystallization from polar organic solvents (DMF/DMSO)	2.15 eV	[34]
**pyrene-*O*-propylamine**	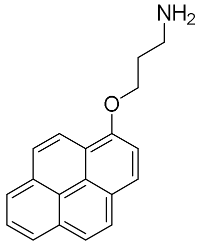	Crystallization from polar organic solvents (DMF/DMSO)	2.19 eV	[34]
**4-chlorobenzylamine**	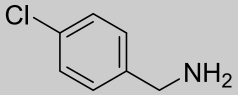	Slow crystallization from HI	1.89 eV	[36]
**3-fluoro-4-chloroanilin**	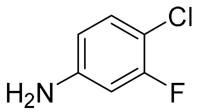	Slow crystallization from HI	1.85 eV	[36]
**3-fluoropiperidine**	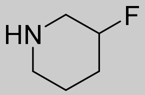	Slow crystallization from HI	1.94 eV	[36]
**4-(aminomethyl)piperidine**	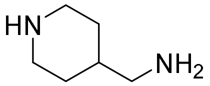	Slow crystallization from HI	1.88 eV	[36]
**histamine**	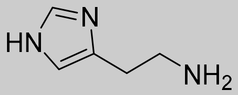	Slow crystallization from HI	1.84 eV	[36]
**4,4,4-trifluorobutan-1-amine**	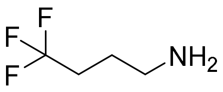	Slow crystallization from HI	1.97 eV	[36]

**(S)-1-(4-chlorophenyl)ethan-1-amine**		Slow crystallization from HI	1.93 eV	[36]
**(S)-1-(4-bromophenyl)ethan-1-amine**		Slow crystallization from HI	1.95 eV	[36]
**(S)-1-(p-tolyl)ethan-1-amine**		Slow crystallization from HI	1.93 eV	[36]
**3,3-difluoropyrrolidine**		Slow crystallization from HI	1.99 eV	[36]
**cyclohexane-1,4-diamine**		Slow crystallization from HI	1.85 eV	[36]
**decylamine**		Rapid precipitation	2.1 eV	this work
**dodecylamine**		Rapid precipitation	2.1 eV	this work
**tetradecylamine**		Rapid precipitation	2.1 eV	this work

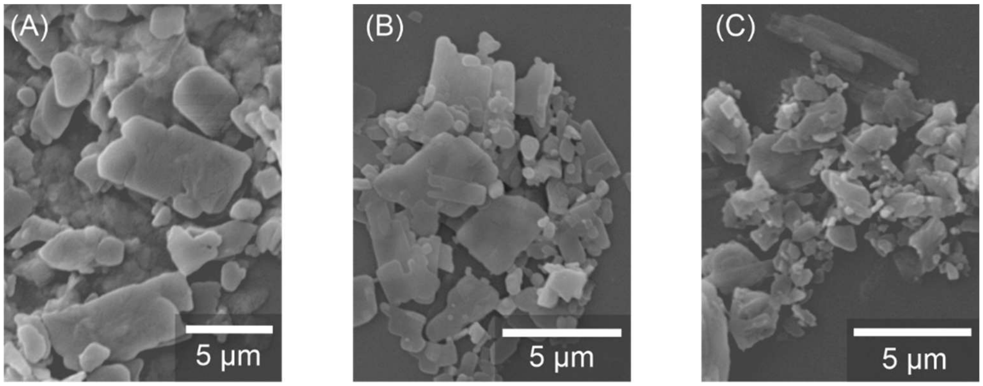

Figure S 1. Scanning electron microscopy images of the powdered C10, C12, and C14 samples

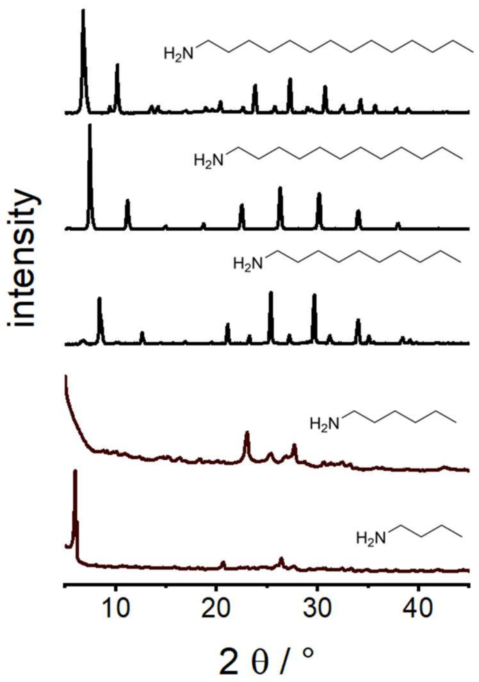

Figure S 2. Powder diffractograms of dropcast product dispersions using different aliphatic amines in the synthesis.

Table S 2. Elemental composition of product silver bismuth iodide powder using various amines as determined by SEM-EDX

**Sample (used amine)**	**Ag (at.%)**	**Bi (at.%)**	**I (at.%)**
**Butylamine**	7.1 (±1.9) %	13.8 (±1.6) %	79.2 (±0.4) %
**Hexylamine**	4.1 (±0.7) %	12.9 (±0.4) %	82.9 (±0.9) %
**Decylamine**	11.8 (±0.5) %	8.9 (±0.1) %	79.3 (±0.4) %
**Dodecylamine**	11.3 (±0.1) %	8.7 (±0.1) %	80.0 (±0.1) %
**Tetradecylamine**	11.3 (±0.1) %	9.7 (±0.5) %	79.0 (±0.6) %
**Fluorophenethylamine**	9.8 (±1.2) %	7.7 (±1.1) %	82.6 (±2.1) %

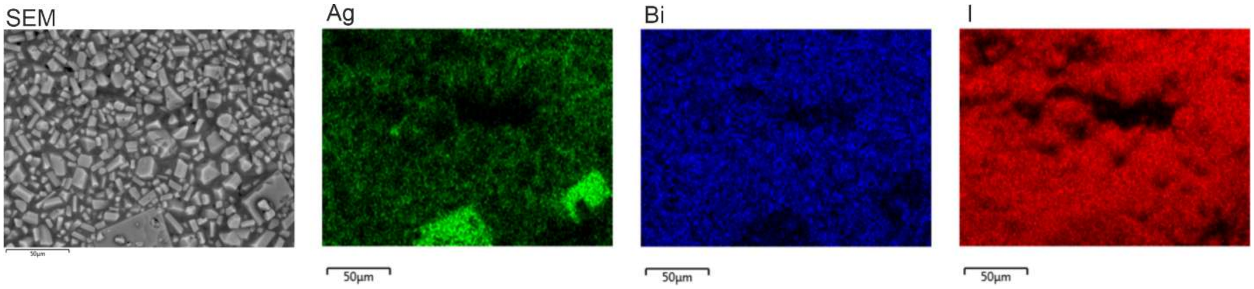

Figure S 3. Scanning electron microscopy overview of silver bismuth iodide synthesized using butylamine and corresponding elemental mapping of Ag, Bi and I by energy-dispersive X-ray spectroscopy.

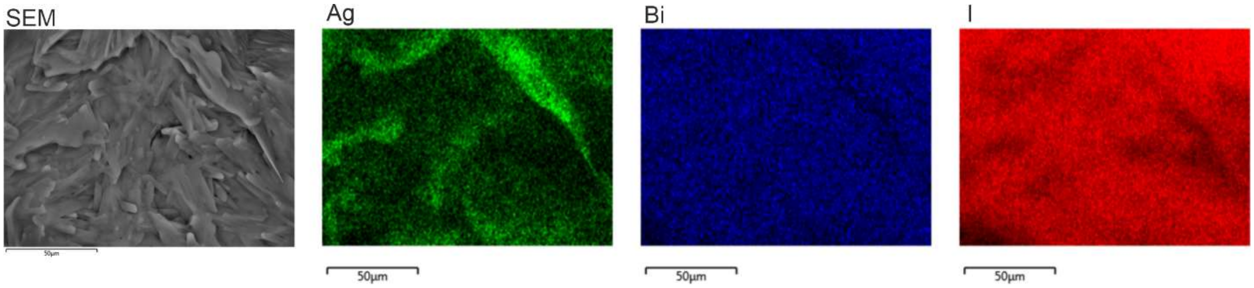

Figure S 4. Scanning electron microscopy overview of silver bismuth iodide synthesized using hexylamine and corresponding elemental mapping of Ag, Bi and I by energy-dispersive X-ray spectroscopy.

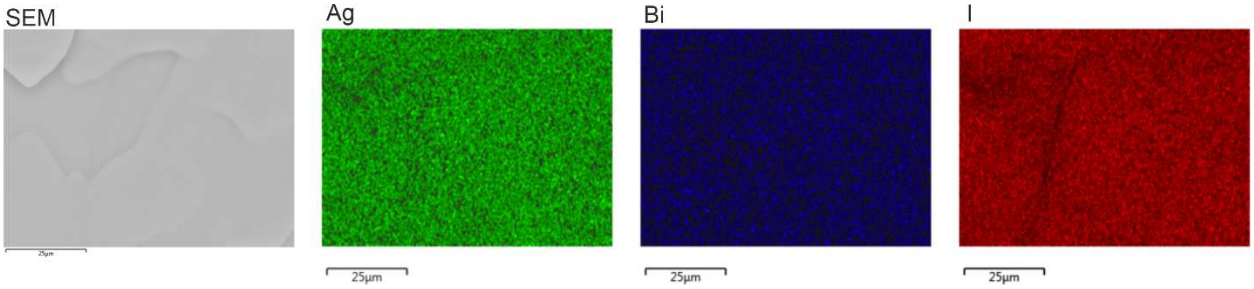

Figure S 5. Scanning electron microscopy overview of silver bismuth iodide synthesized using decylamine and corresponding elemental mapping of Ag, Bi and I by energy-dispersive X-ray spectroscopy.

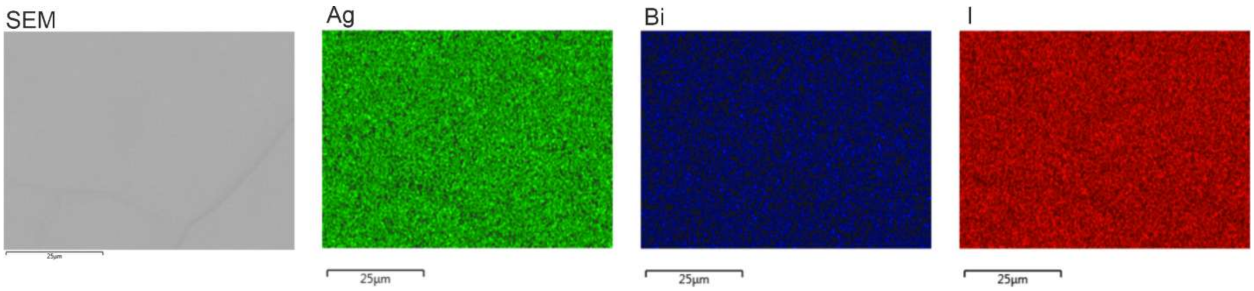

Figure S 6. Scanning electron microscopy overview of silver bismuth iodide synthesized using dodecylamine and corresponding elemental mapping of Ag, Bi and I by energy-dispersive X-ray spectroscopy.

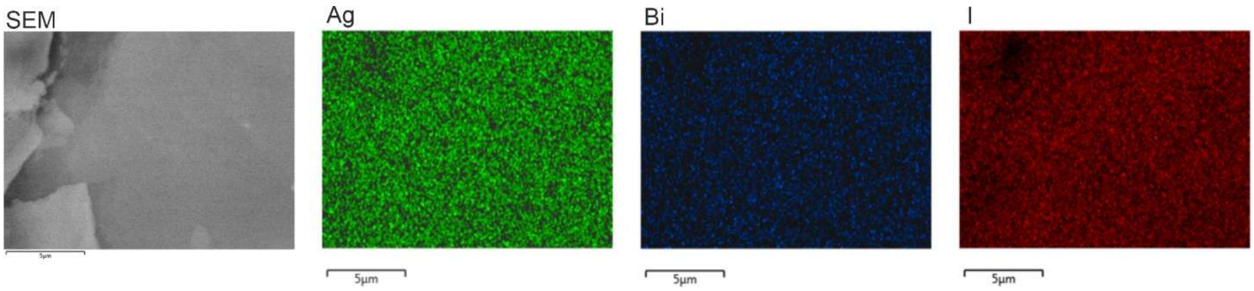

Figure S 7. Scanning electron microscopy overview of silver bismuth iodide synthesized using tetradecylamine and corresponding elemental mapping of Ag, Bi and I by energy-dispersive X-ray spectroscopy.

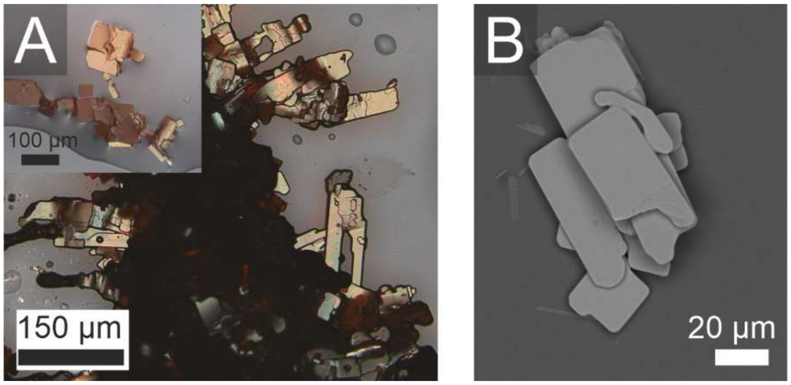

Figure S 8. Micrographs of recrystallized (decylamine)$_4$AgBiI$_8$ in light microscope (left) and scanning electron microscope (right), recrystallized from acetone/hexane mixture.

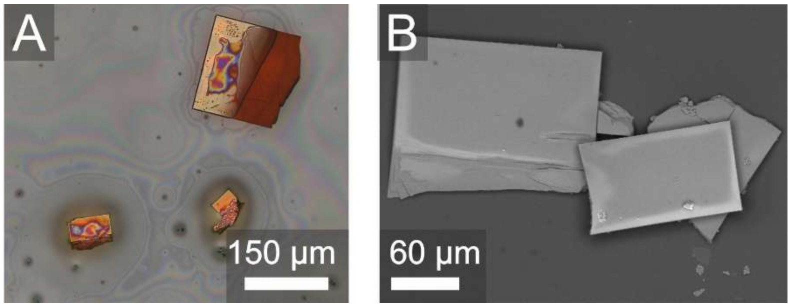

Figure S 9. Micrographs of recrystallized (dodecylamine)$_4$AgBiI$_8$ in light microscope (left) and scanning electron microscope (right), recrystallized from acetone/hexane mixture.

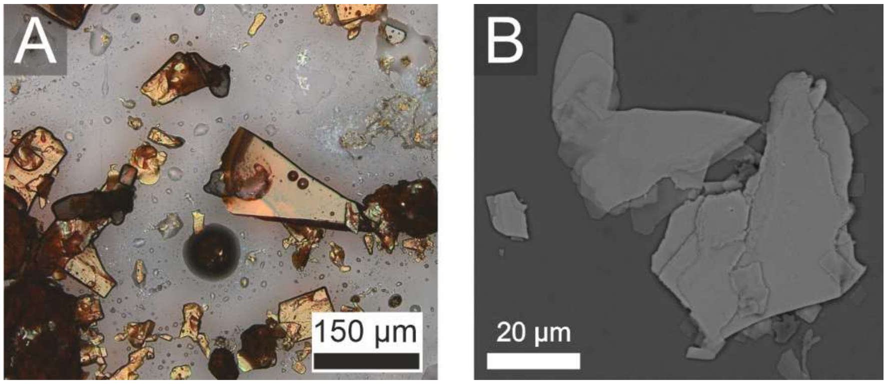

Figure S 10. Micrographs of recrystallized (tetradecylamine)$_4$AgBiI$_8$ in light microscope (left) and scanning electron microscope (right), recrystallized from acetone/hexane mixture.

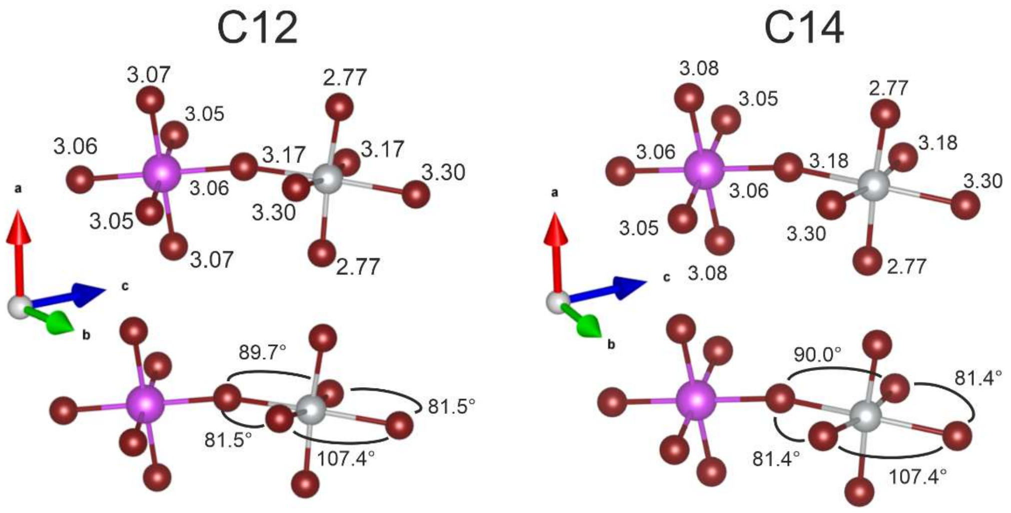

Figure S 11. Coordination of the metal ions in the iodide octahedra as determined by SC-XRD for dodecylammonium (left) and tetradecylammonium (right) $AgBiI_8$ with the bond lengths in Å (top) and the in-plane angles around the Ag cation (bottom).

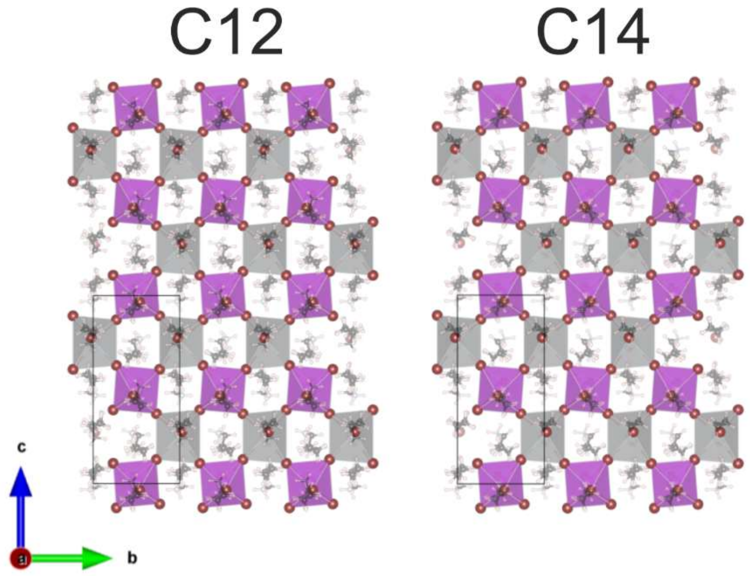

Figure S 12. View along the a-axis of the structures of (dodecylamine)$_4$$AgBiI_8$ (C12, left) and (tetradecylamine)$_4$$AgBiI_8$ (C14, right).

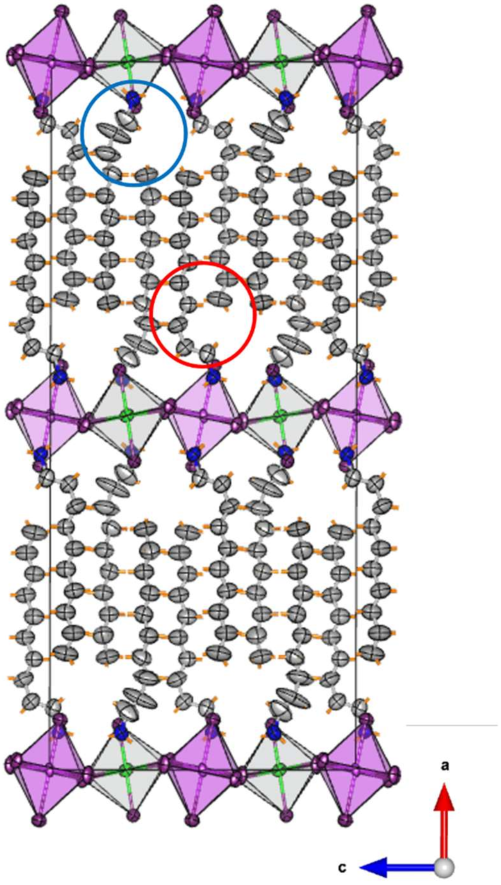

Figure S 13. Structure of C10 viewed along the *b*-axis with atom positions displayed as 50% probability ellipses highlighting two differently rigidly placed amines (marked blue and red). Hydrogen atoms are omitted for clarity

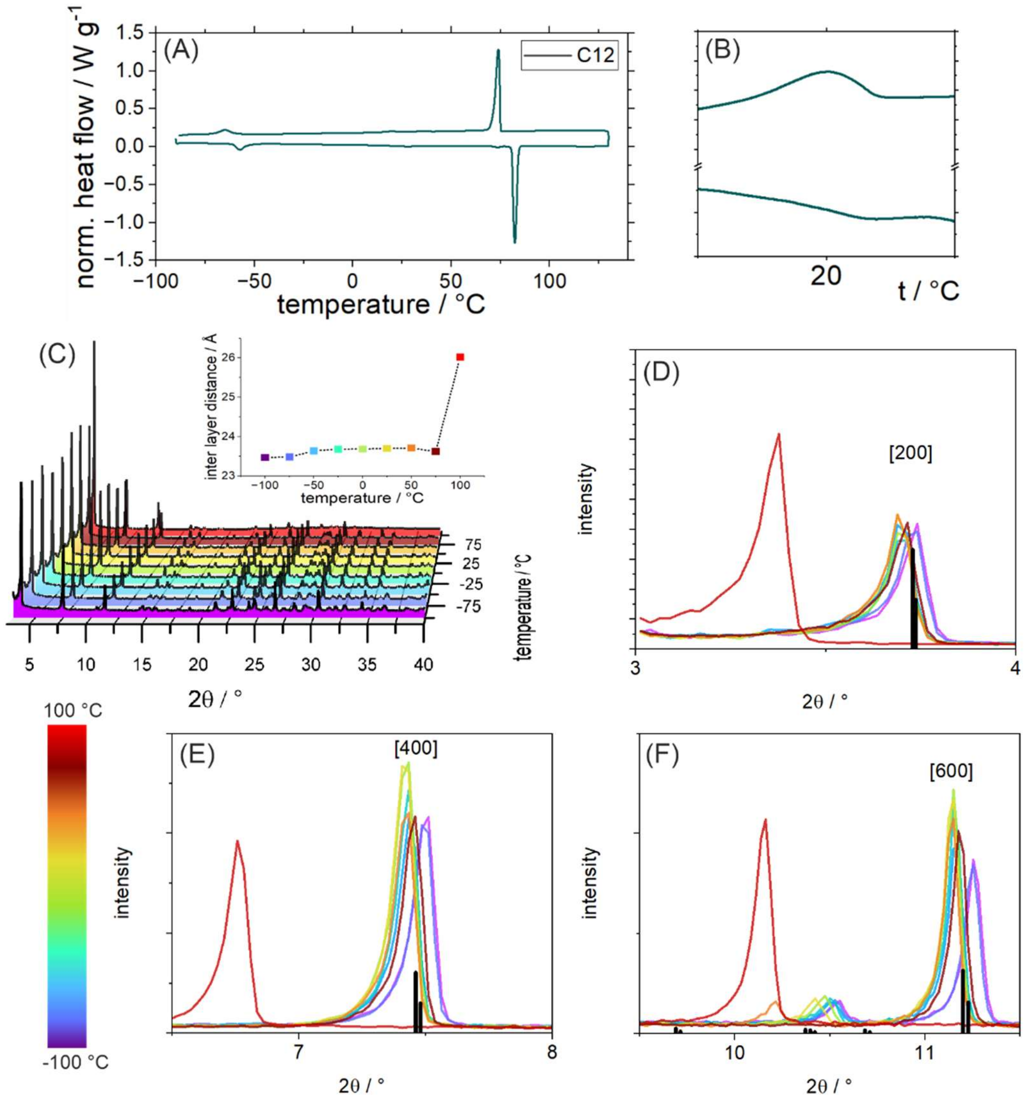

Figure S 14. DSC (A) of the C12 sample, the region around 20°C is shown separate (B) to visualize the small thermal event. p-XRD (C) measured at different temperatures of the C12 sample. The changes at the first 3 observed major reflections corresponding to(D) [200], (E) [400], and (F) [600] are shown in detail with the reference positions calculated from the resolved single crystal structure marked in black

Discussion of temperature dependent measurements

The temperature dependent behavior of the silver bismuth double perovskites at different temperatures was investigated by thermal analysis (differential scanning calorimetry, DSC) and temperature dependent p-XRD. In DSC, multiple reversible thermal effects are apparent around -50 °C, 0°C, and 70°C for the C10 structure (**Figure 15A,B**) and -50 °C, 20 °C and 80°C for the C12 structure (**Figure S16A,B**). The slight shift to higher transition temperatures with increasing chain length matches the chemical expectation as well as the literature reports for similar structures.[24,25] All transitions show a slight hysteresis as the transition temperature is shifting by ca. 10 °C between heating and cooling. Very similar effects can be observed in temperature dependent p-XRD as initially reflections are shifting with the temperature due to minimal changes in the lattice dimensions with temperature. A drastic change is observed above 50 °C where multiple reflections disappear while new ones emerge. This can probably be attributed to a phase transition similar to that reported for the analogue decylamine lead iodide layered perovskite in which the arrangement of the decylamine chain, i.e. the position of the ammonium and its tilt relative to the inorganic layer drastically change, resulting in a 2 Å increase in inter-layer spacing.[25] Notably, the reflections at room temperature and high temperature (100 °C) of the decylamine silver bismuth iodide layered double perovskite reported here match the trend shown for the respective reported room and high temperature decylamine lead iodide layered perovskite patterns (see **Figure S15C**). This suggests similar temperature dependent phase transitions for these two structures. When cooling below room temperature another transition is visible below -50 °C, as evidenced by a sudden shift of the reflection positions, in this case to slightly larger 2θ values, indicating a smaller inter-layer distance and therefore a denser packing of the organic layer. The event which can be seen as weak signal around 5-10 °C in the DSC is not visible in the

temperature dependent diffraction. This is likely due to the similarity of the two structures. In the analogue, known[25] decylammonium PbI_4 perovskite the structural differences for these analogue phase transitions are found to be minimal. This correlates with the small energy difference between the two structures and the accordingly small signal in DSC. Our $AgBiI_8$ sample containing dodecylamine shows overall similar temperature dependent behavior compared to the discussed shorter chain analogue with phase transitions visible above 75 °C and below -50 °C (see **Figure S16**). As expected from the DSC measurement, the transition temperatures appear to be shifted slightly with chain length. The structural changes with a considerably larger inter-layer distance in the high temperature phase and a slight contraction in the low temperature phase also match the behavior of the shorter chain C10 sample. Again, analogues are visible to the reported dodecylammonium PbI_4 structure.[24] The similarity between the $AgBiI_8$ double perovskite structure reported here and the comparable PbI_4 perovskite reference[24,25] in their behavior with temperature is not surprising, and further supports the assumption of such linear chain amines acting as strongly structure directing compounds in the synthesis of the layered double perovskites, contrary to previous assumptions.

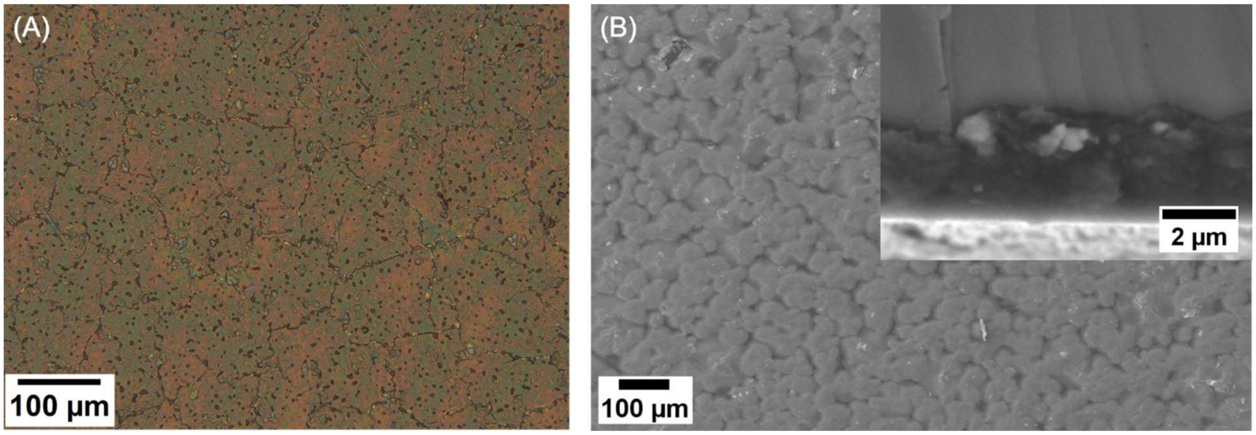

Figure S 15. Thin films of $(C_{12}H_{25}NH_3)_4AgBiI_8$ imaged by light microscopy (A) and scanning electron microscopy (B) in top view. The inset shows the side view of a cut film.

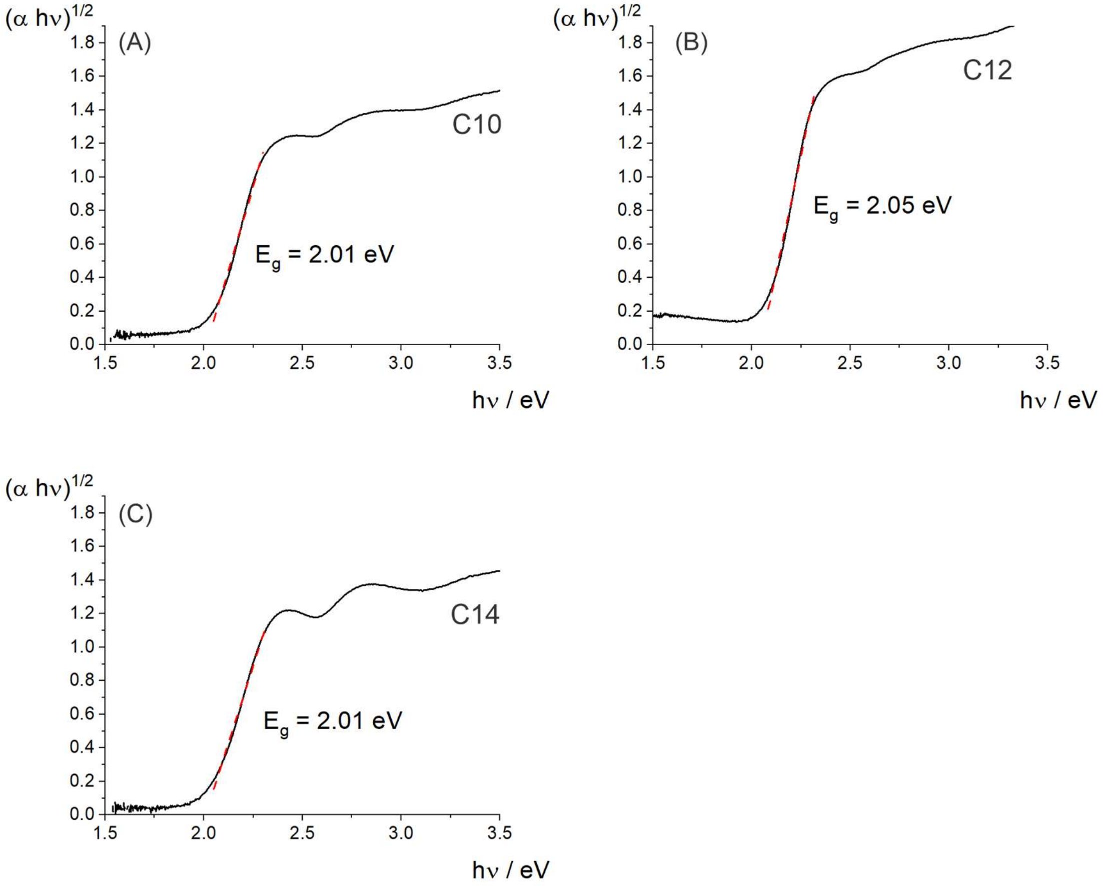

Figure S 16. Tauc plots of the optical spectra of $(C_{10}H_{21}NH_3)_4AgBiI_8$ (A), $(C_{12}H_{25}NH_3)_4AgBiI_8$ (B), and $(C_{14}H_{29}NH_3)_4AgBiI_8$ (C) for the case of a direct band gap with the respective band gap extracted by fitting the linear part of the plot (fitting curve shown in red dashed lines).

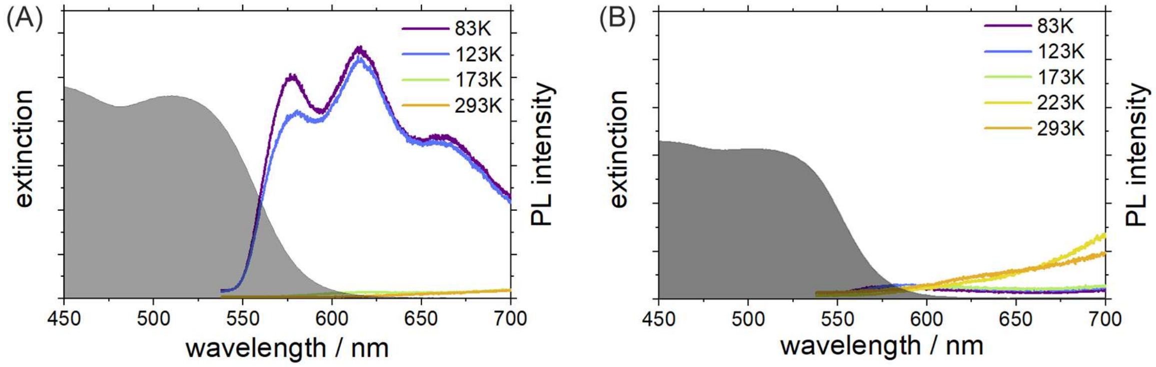

Figure S 17. Temperature dependent photoluminescence spectra of (A) $(C_{10}H_{21}NH_3)_4AgBiI_8$ and (B) $(C_{12}H_{25}NH_3)_4AgBiI_8$ with the corresponding absorption spectra in grey for reference.

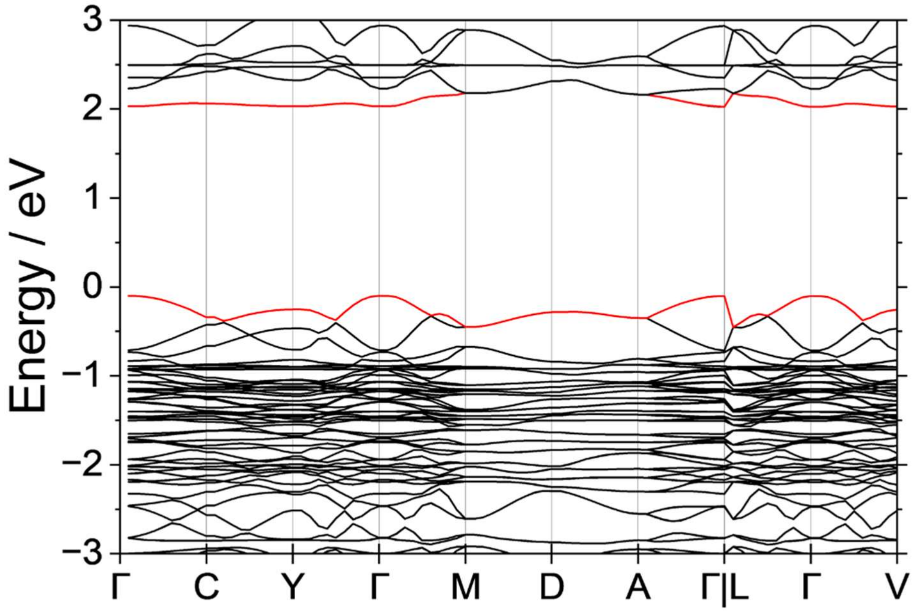

Figure S 18. Calculated band structure of $(C_{12}H_{25}NH_3)_4AgBiI_8$.

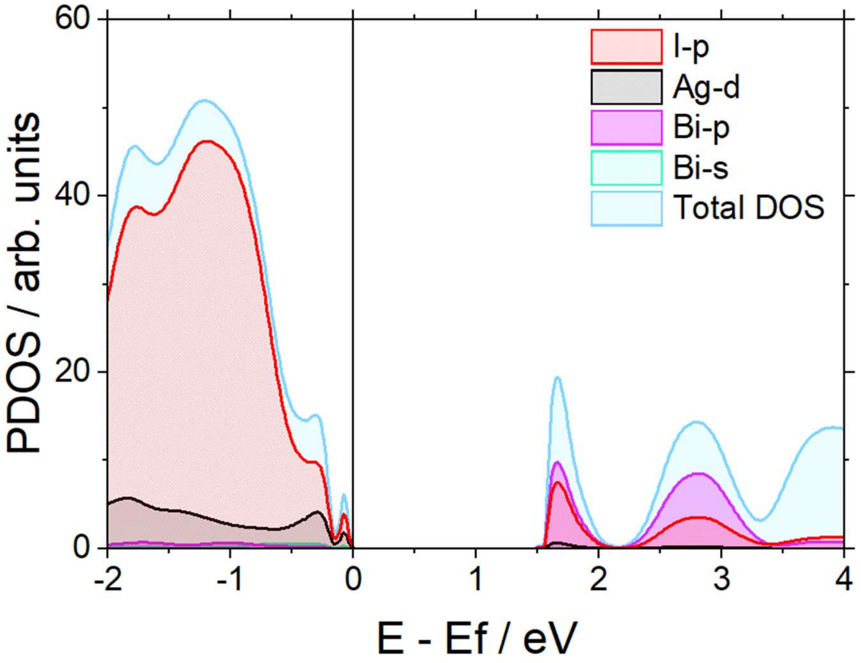

Figure S 19. Density of States of $(C_{12}H_{25}NH_3)_4AgBiI_8$ calculated with spin-orbit coupling.

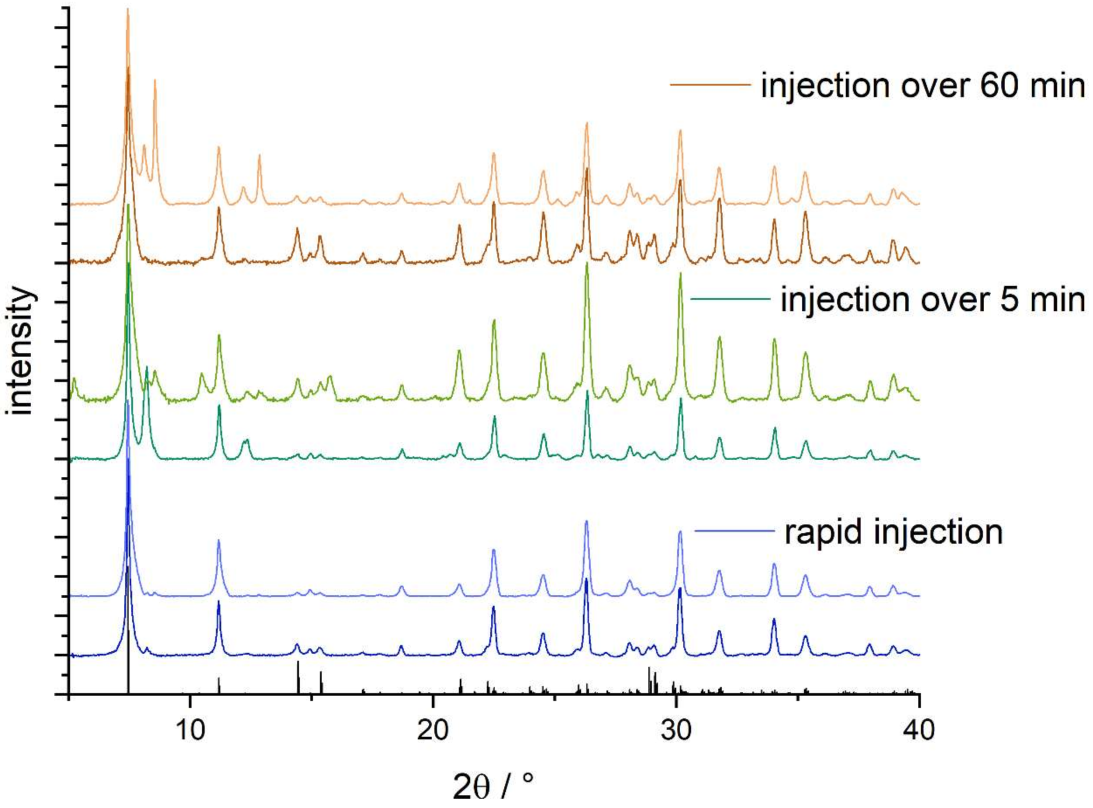

Figure S 20. p-XRD of C12 product obtained through rapid injection (<1 sec, blue traces) and slow addition of the iodide precursor over 5 min (green traces) and 60 min (orange traces) for two syntheses each. All products match the calculated diffraction pattern of $(C_{12}H_{25}NH_3)_4AgBiI_8$ given in black, a gradual addition leads to impurities visible by additional reflections at low 2θ.

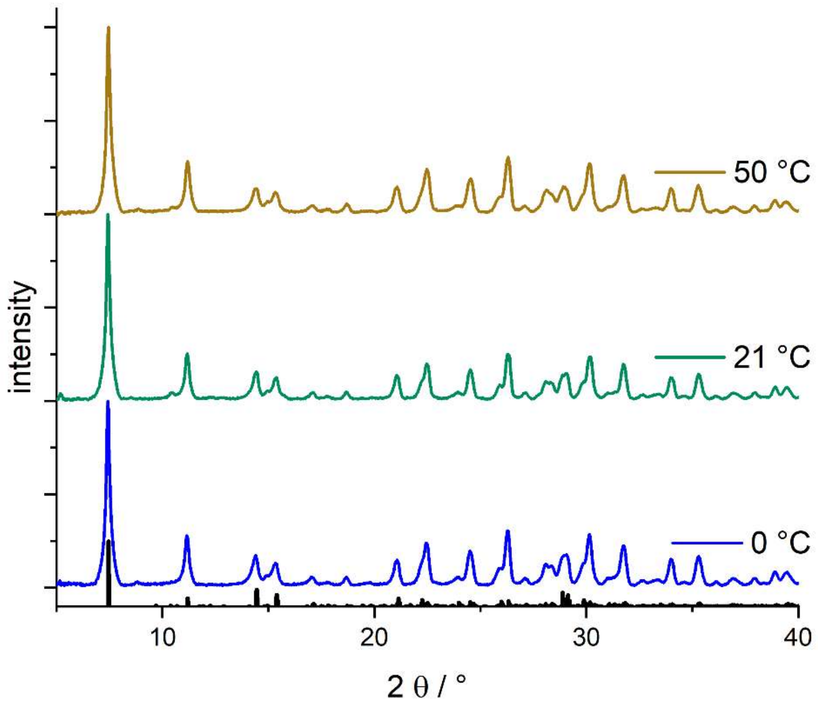

Figure S 21. p-XRD of C12 product obtained through rapid injection at varying temperatures. All products match the calculated diffraction pattern of $(C_{12}H_{25}NH_3)_4AgBiI_8$ given in black.

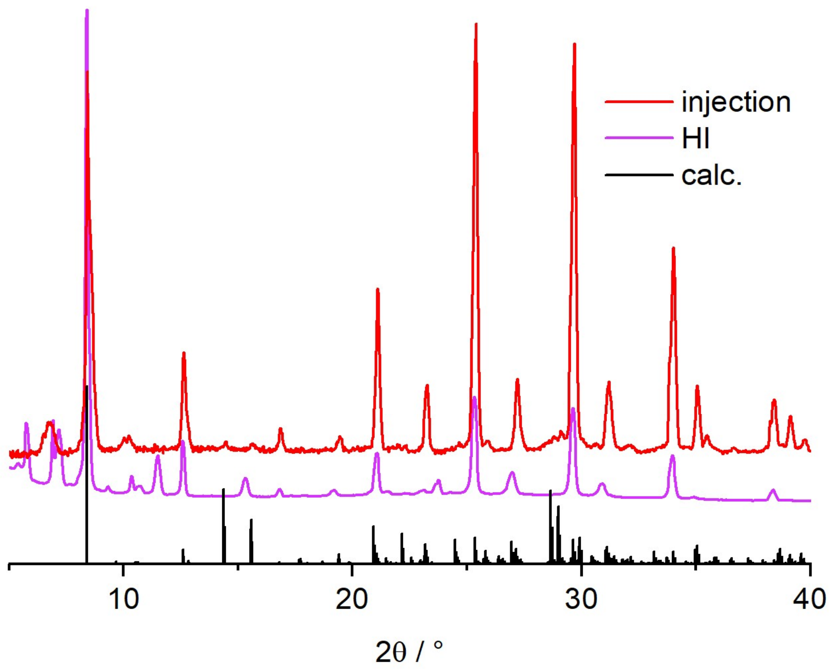

Figure S 22. p-XRD of drop-cast (pref. orientation) C10 product synthesized by fast precipitation after TMS-I injection (red trace) compared to a sample prepared by dissolution of 0.1 mmol AgI, 0.1 mmol BiI_3 and 0.4 mmol decylamine in boiling $HI_{(aq)}$ and slow cooling (purple trace). Both products match the calculated diffraction pattern of $(C_{10}H_{21}NH_3)_4AgBiI_8$ given in black, the HI synthesis has impurities visible by additional reflections at low 2θ.

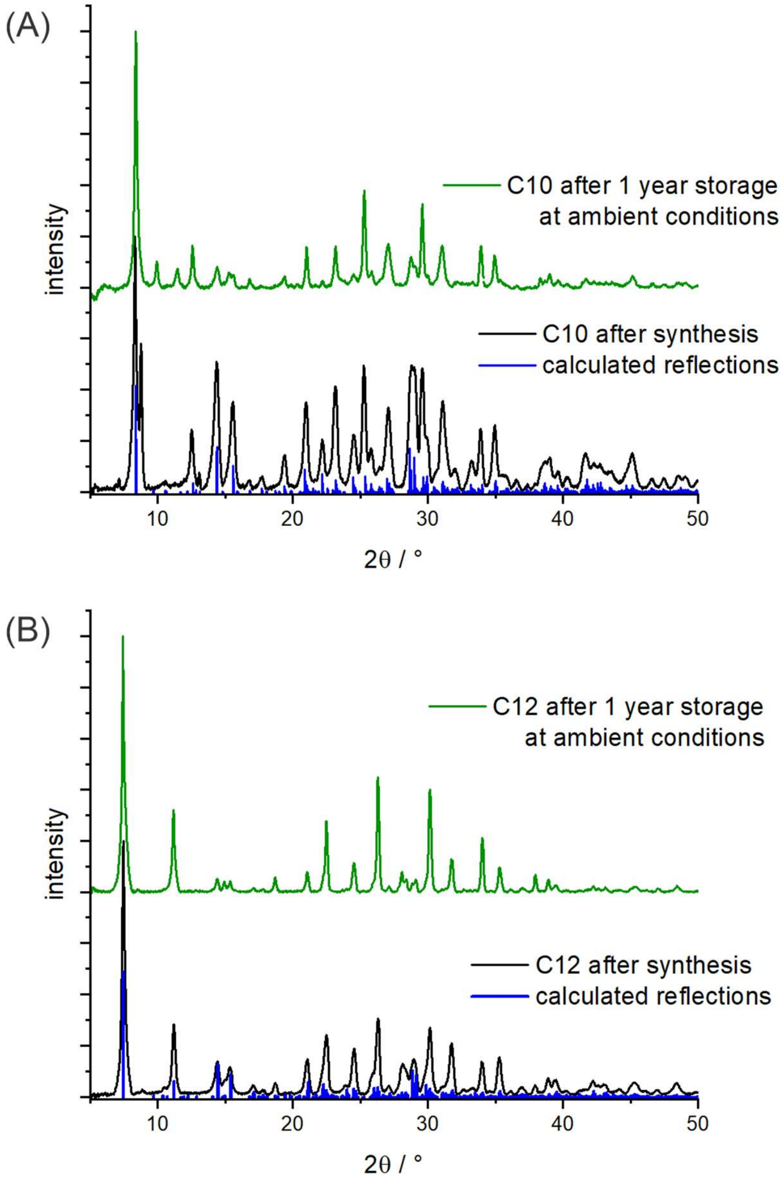

Figure S 23. Powder XRD of $(C_{10}H_{21}NH_3)_4AgBiI_8$ (A) and $(C_{12}H_{25}NH_3)_4AgBiI_8$ (B) after one year storage under ambient atmosphere (green traces) with comparison to the freshly synthesized powder (black traces) and the reflection positions calculated from the resolved single crystal structures (blue lines).

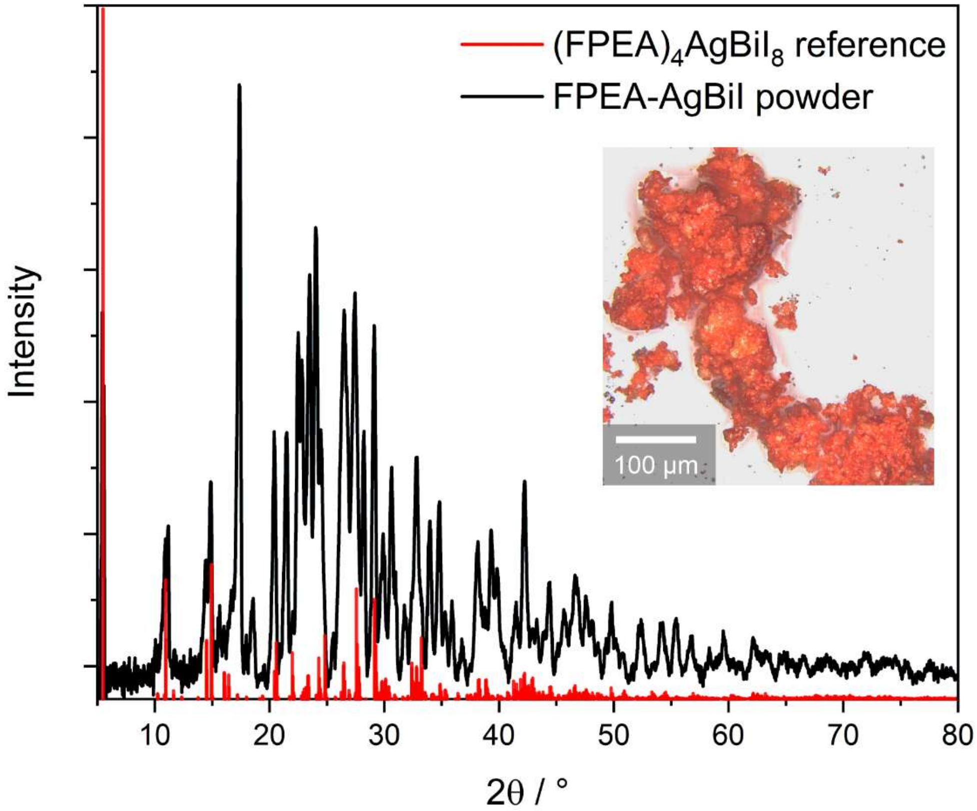

Figure S 24. Powder XRD of layered silver bismuth iodide synthesized by TMS-I injection using 4-fluorophenethylamine with comparison to a literature reference of this material (CCDC 2151233), which was synthesized by slow crystallization from aqueous HI.[19] The inset is showing a micrograph of the product microcrystals.

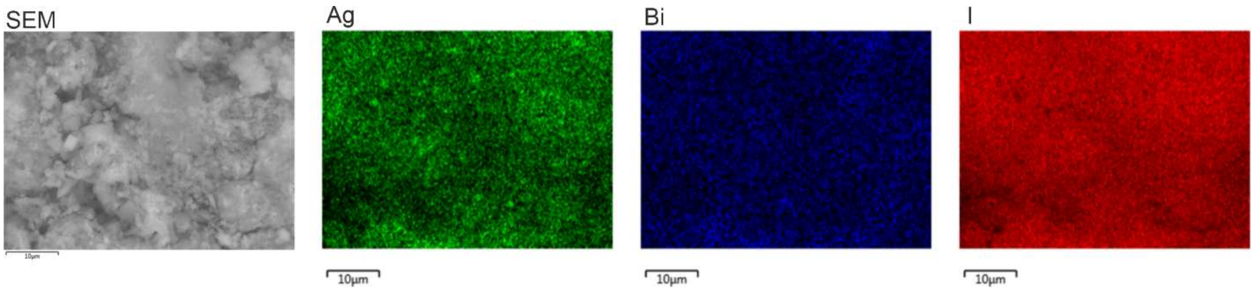

Figure S 25. Scanning electron microscopy overview of silver bismuth iodide synthesized using 4-fluorophenethylamine and corresponding elemental mapping of Ag, Bi and I by energy-dispersive X-ray spectroscopy (quantitative results shown in Table S2 above).

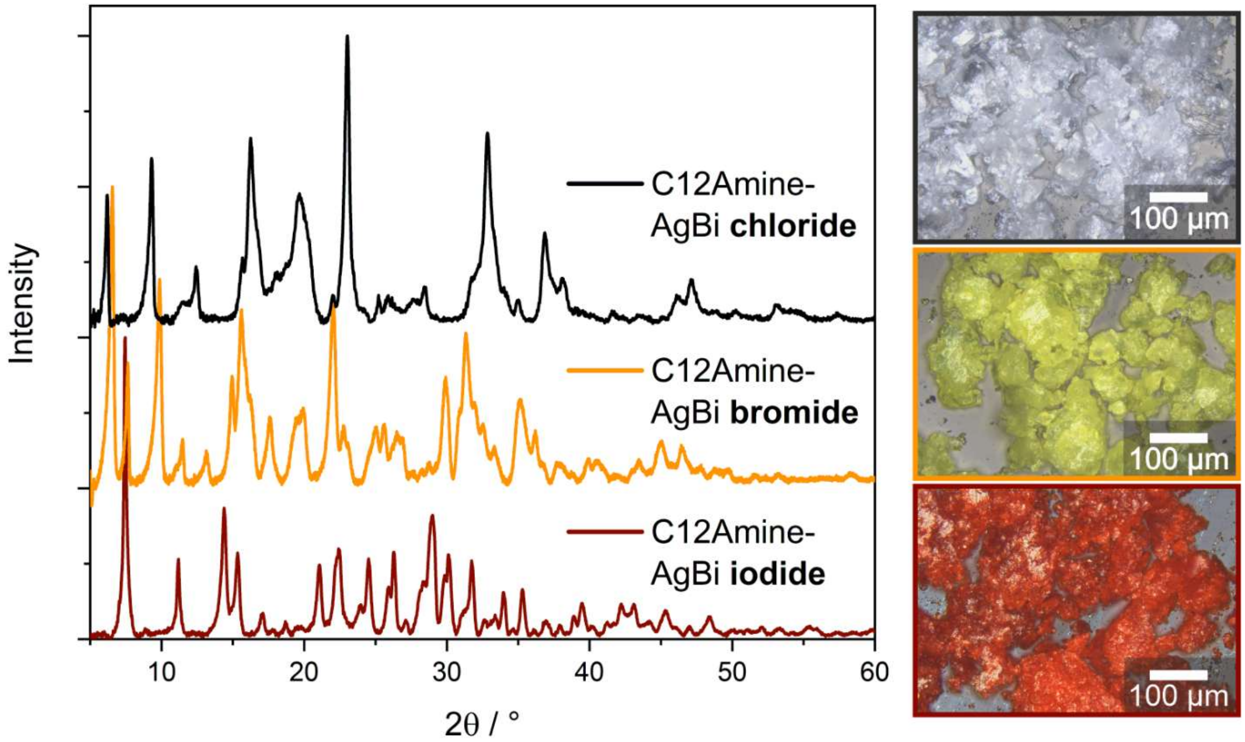

Figure S 26. Powder XRDs of layered silver bismuth halides (from top to bottom chloride, bromide, iodide) synthesized using dodecylamine and micrographs of the corresponding product microcrystals (in the same order top to bottom).

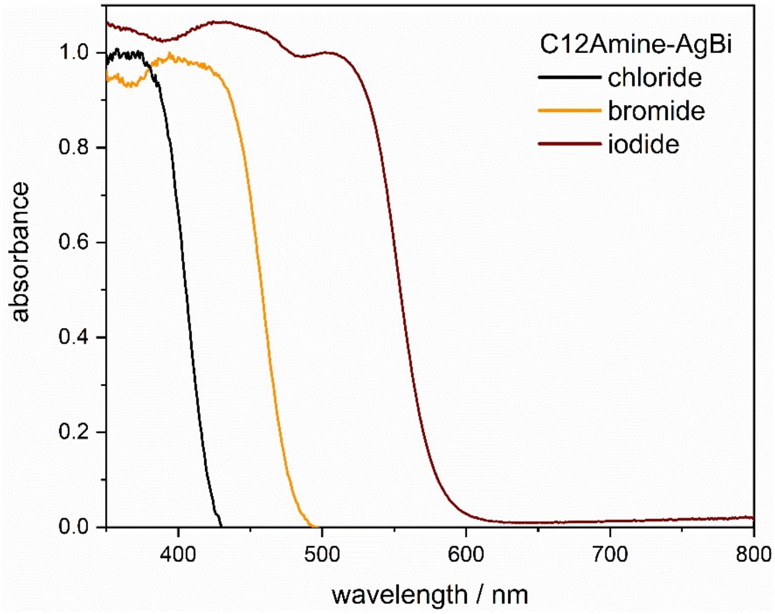

Figure S 27. Absorbance spectra of layered silver bismuth halides synthesized using dodecylamine.
